# Genetic Bottleneck and Panmixia Following the Collapse of a Great Lakes Lake Whitefish Fishery

**DOI:** 10.1111/eva.70337

**Published:** 2026-10-20

**Authors:** Peter T. Euclide, Joseph D. Schmitt, Zak J. Slagle, Jared J. Homola, H. Andrew Cook, Pascal D. Wilkins, Thomas M. MacDougall, Scott T. Koenigbauer, Richard T. Kraus

**Affiliations:** ^1^ Indiana University Bloomington Indiana USA; ^2^ Lake Erie Biological Station, Great Lakes Science Center U.S. Geological Survey Huron Ohio USA; ^3^ Sandusky Fisheries Research Unit Ohio DNR Division of Wildlife Sandusky Ohio USA; ^4^ Department of Fisheries and Wildlife Michigan State University East Lansing Michigan USA; ^5^ Ontario Ministry of Natural Resources Wheatley Ontario Canada; ^6^ Lake Erie Fisheries Unit New York State Department of Environmental Conservation Dunkirk New York USA; ^7^ Ontario Ministry of Natural Resources Port Dover Ontario Canada; ^8^ Department of Forestry and Natural Resources Purdue University West Lafayette Indiana USA

## Abstract

Lake whitefish *Coregonus clupeaformis* abundance has declined throughout most of the Laurentian Great Lakes in North America for two decades. Current management strategies in Lake Erie assume that there is a single stock of lake whitefish that spawns in the West Basin. There is documented lake whitefish spawning activity throughout the lake, leading to questions about locally adapted genetic stocks. Past research on lake whitefish genetic structure in Lake Erie has supported the single‐stock hypothesis, but these studies used only neutral genetic markers or contained a limited number of samples that were collected during the spawning season when stocks would be spatially isolated. Using a GT‐seq panel, we evaluate the genetic structure of lake whitefish collected from spawning shoals in the West, Central, and East basins of Lake Erie and compare these populations to lake whitefish from Lake Huron. We then investigate evidence of local adaptation and conduct demographic reconstruction using whole‐genome sequencing. Our goals were to conclusively quantify intra‐lake genetic structure in Lake Erie and determine whether spawning aggregations possess putatively adaptive variants that could be used for genetic stock identification at a sub‐lake level. Our analysis supported the single‐stock hypothesis in Lake Erie. However, Lake Erie stocks were distinct from neighboring Lake Huron. Whole‐genome analysis did not identify any strong evidence of adaptive loci differentiating stocks. Inference of historic effective population size suggests that declines in lake whitefish populations may have caused a genetic bottleneck. Combined, these results indicate that lake whitefish in Lake Erie may have experienced an Allee Effect after population declines, resulting in a loss of population structure and genetic diversity as spawning shoals were recolonized from a common source.

## 1 Introduction

Inland fisheries around the world have experienced dramatic declines because of overfishing (Allan et al. 2005). Intense harvest in the African Great Lakes has resulted in widespread fish community changes with many unknown consequences to population diversity and genetic structure (Salzburger et al. 2014). Overharvest is often just one stressor to population abundance. In Lake Constance, Europe, two species of lake whitefish (*Coregonus* sp.) have gone extinct due to environmental degradation, and in 2024, the lake whitefish fishery on the remaining species was closed due to major stock declines associated with overharvest (Eckmann and Rbsch 1998; Haase et al. 2025). Fisheries in the North American Laurentian Great Lakes (Great Lakes hereon) are a case study in habitat degradation, overfishing, and climate impacts (Collingsworth et al. 2017; McKenna 2019). Most fisheries in the Great Lakes experienced substantial declines in the late 1800s and early 1900s because of invasive species, habitat destruction, and overharvest associated with increasing industrialization of fishing and fishing gear (Regier et al. 1999). Many species, such as longjaw *Coregonus alpenae* and blackfin Cisco *Coregonus nigripinni*, never recovered (Miller et al. 1989). Other species, like lake trout *Salvelynus namaycush* and walleye *Sander vitreus* , became dependent on stocking throughout much of the Great Lakes (Krueger et al. 1995). While others, such as lake whitefish *Coregonus clupeaformis* , have recovered on their own to varying degrees following protective regulations, habitat restoration, and stocking (Ebener et al. 2008; Schneider and Leach 1977). Fisheries, such as lake whitefish, that declined in the early 1900s have now had 100 years or more of recovery and are entering a new era where the primary stressor is often not overharvest, habitat degradation, or pollution, but instead invasive species and adaptation to shifting temperature and weather regimes (Brander 2010; Collingsworth et al. 2017; Lower et al. 2024). Standing genetic diversity, population structure, and gene flow are all underlying factors that can influence population resilience and adaptability (Chaturvedi et al. 2021; Mason et al. 2020), which remain unquantified for many recovering fisheries. Understanding how populations are structured is important for predicting future impacts of fishery harvest on recovering stocks (Waples et al. 2008). Furthermore, quantifying the consequences of commercial and recreational harvest on population diversity is essential for predicting the adaptive capacity and potential recovery of exploited fisheries (McCairns et al. 2012).

Lake whitefish have consistently supported valuable commercial fisheries throughout the Great Lakes, where basin‐wide harvest has been as high as 22 million pounds per year (Ebener et al. 2021). In Lake Erie, lake whitefish have faced multiple stressors over the last century, including overharvest, eutrophication and sedimentation that degrade spawning habitat, and the introduction of invasive species that prey on lake whitefish larvae. In the Lake Erie basin, lake whitefish were commercially targeted in the early 1800s by seining operations in the Maumee and Detroit rivers, and they declined after the 1890s due to the establishment of extensive pound net, gill net, and trap net fisheries, primarily in Lake Erie's West Basin (Figure 1; Regier et al. 1969). The void in the fishery left by lake whitefish was subsequently filled by cisco *Coregonus artedi* , which were eventually fished to extirpation (Regier et al. 1969). After the collapse of the cisco fishery, efforts shifted back to lake whitefish, with production in Lake Erie peaking in the 1940s at over 5–6 million pounds per year, before collapsing once again in the late 1950s (Baldwin et al. 2018). Lake whitefish catches did not improve again until the 1980s, and they continue to support limited commercial harvest of around 1 million pounds per year in Lake Erie (MacDougall et al. 2023). Despite twice recovering on their own after fisheries‐driven collapses, there is evidence that present‐day lake whitefish populations in Lake Erie are a fraction of what they were historically (Ebener et al. 2021; Oldenburg et al. 2007). Furthermore, recruitment of lake whitefish is variable and is undoubtedly lower than it was historically in western Lake Erie and the Detroit River (Ebener et al. 2021; Roseman et al. 2007). In response, there are ongoing efforts throughout the Great Lakes, particularly the Detroit River, to restore spawning habitat for lithophilic spawners like lake whitefish (Fischer et al. 2018; Kalejs et al. 2022). Some studies also suggest that there may be high‐quality spawning habitat in areas that are not currently closely monitored by fisheries managers (Amidon et al. 2024; Oldenburg et al. 2007; Schaefer et al. 2021). These factors have led to questions about the remaining genetic diversity following two major declines, given the substantial demographic impacts that population crashes can have on effective population size and intraspecific diversity (e.g., Andrews et al. 2025). Furthermore, given that widespread spawning may be occurring in Lake Erie, conclusive genetic evidence of spawning stock structure is desired to evaluate the single‐stock management assumption driving policy in Lake Erie (Amidon et al. 2024; Schaefer et al. 2022).

**FIGURE 1:**
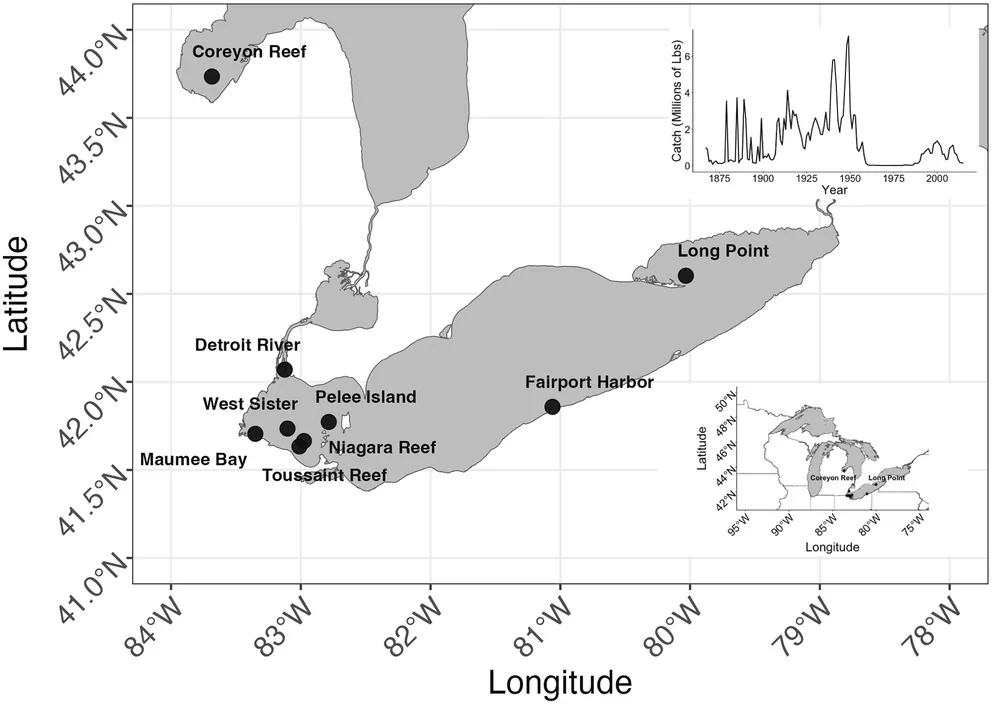
Sample locations of all lake whitefish collected for sequencing. All fish were collected during the fall, during or just before spawning season, and therefore were presumed to be close to their final spawning location. Fish were assessed for maturity, and in all cases, at least some of the collected fish were gravid at the time of their collection, further indicating that spawning aggregations were being sampled. The inset in the top right shows the annual harvest of lake whitefish in Lake Erie in millions of pounds (Lbs) reported from 1867 to 2015.

In many large lakes, lake whitefish exhibit complex population genetic structure, and even sympatrically evolved ecotypic diversity (e.g., Hénault et al. 2026; Mérot et al. 2023; Stott et al. 2010, 2004; Vuorinen et al. 1993). Furthermore, as a genus, *Coregonus* has proven to be highly adaptable, often expressing high levels of fine‐scale intraspecific diversity, genetic structure, and local adaptation (Blain et al. 2025). In Lake Michigan, lake whitefish exhibit complex spawning stock structure in the northern portion of the lake, where six genetic management units have been proposed (Nathan et al. 2016; Shi et al. 2022; VanDeHey et al. 2009). This population structure is geographically associated and appears to be the result of natal homing and local adaptation. Multiple islands of divergence were discovered in the genome that differentiate geographically adjacent populations, suggesting that lake whitefish may be locally adapted to their natal spawning sites (Shi et al. 2022). Because lake whitefish in Lake Michigan appear to express spawning site fidelity, spatial structure is now being considered during fishery surveys and when establishing harvest regulations for Lake Huron and Lake Michigan (Izzo et al. 2024; Shi et al. 2022). While the lake whitefish fishery is largely managed at the local level, there is also clear genetic differentiation among the Great Lakes, based on other genetic studies suggesting significant species‐level diversity exists within the Great Lakes (Graham et al. 2022; Stott et al. 2024, 2013, 2004). This begs the question of whether cryptic intraspecific diversity is being overlooked by managing Lake Erie as a single stock.

Recent evidence suggests that there is substantial spawning and early recruitment (i.e., eggs and larvae) of lake whitefish across all basins of Lake Erie (Amidon et al. 2024; Schaefer et al. 2022). Furthermore, ongoing restoration efforts in the Detroit River and other locations of the lake have reopened habitats for lake whitefish to spawn (Roseman et al. 2007; Stott et al. 2024). These spawning sites are presumed to have been recolonized by Lake Erie lake whitefish, but this remains unclear. This new spawning calls into question the single‐stock hypothesis. Preliminary studies found that there was some evidence, albeit limited, of genetic structure localized in islands of divergence distributed throughout the genome among individuals collected during the summer and fall in Lake Erie (*n* = 88; Euclide et al. 2022). However, this study was limited because most fish were captured outside of the spawning period, which could result in missing subtle genetic differences associated with spatially disjunct spawning aggregations that maintain isolation via spawning site philopatry (Euclide et al. 2022; Stott et al. 2024). Therefore, collections of lake whitefish genetic material during the spawn were needed to further elucidate whether Lake Erie lake whitefish are comprised of genetically unique subpopulations. The goals of the present study were to: (1) determine whether lake whitefish spawning aggregations are genetically distinct from one another using molecular data from the Detroit River, West Basin, Central Basin, and East Basin of Lake Erie, and Saginaw Bay, Lake Huron, and (2) to determine whether lake whitefish spawning aggregations have evidence of locally adapted genes associated with geographic regions of the lake, and (3) quantify past and present genetic diversity within Lake Erie to investigate how historic fluctuations in population size may have altered the genetic diversity and adaptability of the Lake Erie lake whitefish population.

## 2 Methods

### 2.1 System Description and Sampling Design

Lake Erie is a large lake (surface area ~26,000 km^2^) composed of three basins: the West Basin, which is shallow (3–7 m deep on average) containing many islands, rocky reefs, and major rivers and river deltas used by fish for spawning. The Central Basin, which is deeper (15–18 m) and contains limited physical structure, experiences long periods of anoxia in the late summer and fall (Stow et al. 2023). Finally, the East Basin, which is the deepest portion of the lake (15–25 m), therefore, contains the most sub‐thermocline habitat during the summer and contains many small reefs and river inputs used by fish for spawning. Lake whitefish have been shown to use West Basin reefs extensively to spawn, and egg and larval fish sampling indicates that significant recruitment occurs in these areas (Amidon et al. 2024, 2021a). However, lake whitefish eggs and larvae have also been found in the Detroit River (Roseman et al. 2007), the Central Basin (Amidon et al. 2024, 2021a, 2021b), and the East Basin, albeit in lower concentrations than the West Basin.

The primary goal of the present study was to collect and analyze lake whitefish genetic material from the recently reestablished populations in the Detroit River and those in all three basins of Lake Erie. Some of the lake whitefish in the collection were captured by commercial fishing operations (e.g., Maumee Bay, Ohio and Long Point Bay, Ontario), whereas others were intentionally targeted by USGS fisheries scientists using 3.5″ and 4.5″ stretch mesh gillnets, which were 500′ long and had 8′ panel depth, and were set on the bottom (West Basin reefs including Niagara, Toussaint, Detroit River, Fairport Harbor). Nets were deployed in areas with known spawning shoals or in areas where eggs and larvae had been captured in the past, and all sampling took place in the late fall when lake whitefish are forming spawning aggregations. In some areas, collections required just a few overnight sets (e.g., Niagara Reef, Toussaint Reef), whereas other areas required extensive effort and yielded few fish (USGS sampling in Detroit River and Fairport Harbor, Ohio), suggesting that the relative densities in these areas are low (Figure S1; Table S1). Some fish were captured via boat electrofishing during an acoustic telemetry tagging operation inside the Detroit River by Ontario Ministry of Natural Resources (OMNR) scientists. Whenever possible, fish were sexed, assessed for reproductive state, and total length was measured to the nearest millimeter. An approximately 1 cm^2^ fin clip was collected from the caudal fin of each fish and stored in 90% ethanol for DNA extraction. Samples collected by OMNR at Pelee Island were sampled in conjunction with an acoustic telemetry study targeting spawning stocks, which sampled fish using 500′ monofilament experimental gillnets with 4″, 4.5″, 5″, 5.5″, and 6″ stretch mesh. Nets were set overnight in 4–6 m of water.

Because past studies have found very limited population structure within Lake Erie, an outgroup containing samples from a population that is related but still presumed to be genetically differentiated from all Lake Erie populations was included in the analysis to contextualize results. The purpose of the outgroup was threefold: first, the samples were to provide a baseline estimate of what population structure looks like at the between‐lake level within the Great Lakes that could be compared to any structure observed within Lake Erie; second, these samples were to be used to quantify any potential adaptive variation that existed between Lake Erie as a whole and the neighboring Lake Huron lake whitefish population and to again contextualize any outliers found within Lake Erie; third, because Lake Huron has experienced similar system‐wide impacts to their fishery, by conducting demographic reconstruction on both the target population and outgroup we aimed to identify consistencies and discrepancies between the two systems. Therefore, samples of 15 adult lake whitefish that were collected via gillnet surveys conducted in Saginaw Bay, Lake Huron, during the fall spawning period were obtained and used as this outgroup and included in the analysis (Koenigbauer et al. 2026).

### 2.2 Sequencing Methods

In Lake Michigan, genetic stock assessments are now being conducted using a genetic genotyping‐in‐thousands (GT‐seq) genotyping panel (Krause 2025). To quantify the population structure in Lake Erie across neutral loci (Objective 1), we conducted a spatially broad survey using this GT‐seq panel for all individuals collected across Lake Erie and Saginaw Bay, Lake Huron. GT‐seq panels contain a set of predetermined loci that allow for direct comparison in future meta‐analyses to other studies, making it important to collect these data when possible. Furthermore, due to cost limitations, approximately twice as many samples and two additional collection locations were able to be surveyed using GT‐seq compared to a whole‐genome sequencing (WGS) survey (Table 1). However, because GT‐seq panels are susceptible to ascertainment bias, wherein loci informative for one set of populations may not be relevant elsewhere, thereby misleading conclusions (Lachance and Tishkoff 2013; Malomane et al. 2018), comparing results of the GT‐seq to WGS was used as corroborating evidence for population structure observed using the GT‐seq panel. The WGS survey was conducted on a subset of individuals collected from Lake Erie and Saginaw Bay to complete Objectives 2 and 3 and overlapped directly with samples used in the GT‐seq survey. This second dataset contained fewer individuals and collection sites, but a much higher density of markers that were randomly distributed across the genome. This dataset was therefore primarily used to quantify demographic history and identify putative outlier loci that differentiated among collections.

**TABLE 1:** Sample size and diversity statistics for GT‐seq and whole‐genome sequencing results. Regions indicate the general location of the collection, while the Collection refers to the specific sample location where collections were made. Sample size (*N*) differed for GT‐seq and whole‐genome sequencing. Observed (Ho) and expected (He), and inbreeding coefficient (*F*_IS_) are defined as weighted means. The total number of private alleles (PA) is rarefaction‐corrected by sample size.

Region	Collection	GT‐seq					Whole genome				
		*N*	Weighted mean Ho	Weighted mean He	Total PA corrected	Weighted mean *F*_IS_	*N*	Weighted mean Ho	Weighted mean He	Total PA corrected	Weighted mean *F*_IS_
*L. Huron* , Saginaw Bay	Coreyon Reef	15	0.27	0.25	11.25	−0.03	15	0.15	0.12	6125.71	−0.22
L. Erie, West Basin	Detroit River	11	0.25	0.23	1.53	−0.02	8	0.13	0.11	3747.18	−0.15
—	Maumee Bay	134	0.27	0.25	1.33	−0.08	20	0.15	0.12	4986.43	−0.23
—	Niagara Reef	23	0.26	0.25	0.75	−0.04	23	0.15	0.12	4551.55	−0.22
—	Pelee Island	73	0.25	0.24	1.92	0	0	—	—	—	—
—	Toussaint Reef	15	0.28	0.24	1.6	−0.11	15	0.15	0.12	4485.39	−0.25
—	West Sister	14	0.25	0.24	1.72	0.01	0	—	—	—	
L. Erie, Central Basin	Fairport Harbor	6	0.26	0.22	0	−0.05	6	0.15	0.12	5.04	−0.15
L. Erie, East Basin	Long Point	40	0.28	0.26	1.67	−0.08	40	0.16	0.13	7165.76	−0.21

### 2.3 GT‐Seq

DNA extraction from all 371 lake whitefish fin clips was performed using either QIAGEN DNeasy DNA extraction kits following the manufacturer's protocol or a chelating resin‐based procedure modified from Baumsteiger and Kerby (2009). For chelating resin extractions, fin clips were subsampled using a 4‐mm biopsy punch, incubated overnight in 7% Chelex, heated at 95°C for 15 min, cooled to 4°C for 2 min, then centrifuged at 2200 × *g* (rcf) for 5 min before pipetting off the supernatant.

Sequencing library preparation followed the GT‐seq approach described in Bootsma et al. (2020) using the lake whitefish GT‐seq panel developed by Krause (2025). The GT‐seq panel amplifies 471 single‐nucleotide polymorphisms (SNPs) across 437 amplicons. Samples were amplified individually using 19.2 μL of pooled primers and 44.8 μL Qiagen Plus master mix. PCR products were diluted in a 96‐well plate at a rate of 3 μL product to 37 μL nuclease‐free water. Next, plate or sample‐specific barcodes were added to allow for multiplexing. Sample‐specific product concentrations were standardized using a SequalPrep normalization plate before pooling samples into a single library per plate. Library concentrations were then verified using a Qubit fluorometer, and the quality of the libraries was assessed using agarose gel electrophoresis. Sequencing was performed on either the Illumina Novaseq X‐plus or MiSeq sequencing platforms using 2 × 150 bp flowcells at the University of Wisconsin Biotechnology Center, Madison, Wisconsin.

Raw sequencing reads were processed using the GTscore pipeline in R (McKinney et al. 2020). Amplicon counts were generated using AmpliconReadCounter.pl. with species‐ and panel‐specific primer‐probe files. Single‐nucleotide polymorphisms (SNPs) were genotyped using GTscore's polyGen genotyping algorithm based on locus and allele read count tables. Loci with genotyping success rates below 0.80 were excluded from analysis using GTscore filters. Genotypes were exported in genepop format, then read into R using the package adegenet (Jombart 2008). The poppr package (Kamvar et al. 2015) was used to remove samples with genotyping rates < 0.7. The resulting dataset was used for population genetic analyses described below.

### 2.4 Whole‐Genome Sequencing

Extracted DNA from 127 lake whitefish spanning four West Basin spawning sites, one Central Basin spawning site (Fairport Harbor), one East Basin spawning site (Long Point), and one Lake Huron outgroup (Koenigbauer et al. 2026) were submitted for library preparation and whole‐genome sequencing (WGS) at the Purdue Genomics and Genome Editing Facility at Purdue University, West Lafayette, Indiana. These samples were subsetted at random from the full GT‐seq dataset. At the core, individual PE 150 bp read‐length Illumina libraries were constructed using a DNA Nano kit (Illumina) and then sequenced to a target depth of coverage of 15× using six sequencing lanes on a NovaSeq Xplus 25B flowcell. This generated approximately 20 billion basepairs. Following assessment of lane clustering, one additional lane of sequencing was conducted on a subset of individuals with low clustering, which produced an additional 3.1 billion basepairs. Illumina adapters were removed, and the leading and trailing 20 bp of each read were dropped as well as any read with a length < 30 bp, using trimmomatic (Bolger et al. 2014).

The trimmed data were then aligned to the dwarf lake whitefish genome (NCBI genome assembly: ASM2061545v1; Mérot et al. 2023) using the BWA mem algorithm (bwa v. 0.7.17; Li 2013). Aligned reads were then converted into a VCF genotype file following the GATK4 best practices (GATK4 v. 4.6.0.0; Van der Auwera and O'Connor 2020; Van der Auwera et al. 2013). Resulting VCF files were hard filtered using the VarientFiltration module of GATK4 to first select only SNP variants and then flagging all genotypes with QD < 2, SOR > 3, FS > 60, MQ < 40, MQRankSum < 12.5, or ReadPosRankSum < −8. Hard‐filtered variants were further filtered for quality using VCFtools (Danecek et al. 2011) to remove SNPs that failed GATK4 filtering parameters, had excessively low or high depth of coverage, had a minor allele count of < 3, or were not biallelic. Following these initial filters, potential paralogs were identified using ParaMask, run separately for each chromosome. ParaMask uses a three‐step process to identify putative paralogous SNPs and regions based on genotype sequencing depth and read‐ratio deviations, and positional clustering along the chromosome (Tjeng et al. 2025). First, potential multi‐copy regions are identified using an expectation–maximization approach to identify and mark variants with usually high heterozygosity. Next, multi‐copy SNPs are reclassified using read‐ratio deviations, which can find regions often missed by heterozygosity excess approaches. Finally, multi‐copy haplotypes are identified using a clustering approach that groups multi‐copy variants into paralogous regions based on chromosomal position. All SNPs identified with a final classification of potentially multi‐copy in ParaMask were removed from further analysis. Finally, all loci with a genotyping rate of < 85% were removed. Two datasets were then created: one for population genetic assessments that was pruned for linkage disequilibrium using PLINK2 (Purcell et al. 2007) and a second containing all the retained variants used for assessment of adaptive variation and demographic history. A full summary of filtering parameters and variant quality control can be found in Table S2.

### 2.5 Population Genetic Analysis

Population genetic structure and diversity were assessed primarily using the GT‐seq dataset, and patterns were corroborated using the WGS dataset with SNPr v. 1.2.14 (Hemstrom and Jones 2023). In both cases, broad patterns of population structure were dissected using principal component analysis (PCA) and then visualized using biplots generated using ggplot2 and ggsci packages (R Core Team 2025; Wickham 2009; Xiao 2018). Two separate PCAs were conducted for each dataset, once using all individuals and a second time using only individuals from Lake Erie. Pairwise genetic distance among collections was calculated as weighted‐mean pairwise Weir and Cockerham *F*_ST_ (Weir and Cockerham 1984) using SNPr (Hemstrom and Jones 2023) and the *calc_pairwise_fst()* function with collection areas as the facet. Observed and expected heterozygosity, the number of private alleles, and bootstrapped estimates of inbreeding coefficient (*F*_IS_; 99 permutations) were calculated for each locus (variant and invariant) and population, also using SNPr and the *calc_ho()*, *calc_het()*, *calc_private()*, and *calc_fis()* functions (Hemstrom and Jones 2023).

### 2.6 Demographic History

The last 150 generations of effective population size (*N*_e_) were reconstructed using GONE2 and the WGS dataset (Santiago et al. 2020, 2025; Downloaded July 23, 2025). This approach uses linkage disequilibrium among reference‐aligned SNP loci to estimate how effective population size has changed over the last 100–200 generations. GONE2 was run separately for all individuals from Lake Erie (*N* = 112) and Coreyon Reef, Lake Huron (*N* = 15), with the assumption that they are both panmictic populations with limited structure. We ran GONE2 using 10 different random sets of 500,000 loci for each population for 20 total simulations. Rolling averages across the 10 replicates were calculated for 10 generation windows in R. Breakpoints in the rolling average were identified using the *breakpoints()* function from the strucchange package v. 1.5‐4 in R, and the number of changes set was identified by looking at the change in BIC and identifying a plateau. 95% confidence intervals around each breakpoint were then estimated using the *confint()* function and used to report approximate locations of breakpoints in generations before present. This approach detects inflection points where the mean or regression coefficient changes by dividing the time series into segments and identifying the set of break locations that minimize error (Zeileis et al. 2002, 2003). Therefore, changepoint analysis was used to estimate the onset time in generations before present when the estimated *N*_e_ either increased or decreased compared to prior generations. To assign dates to generations before present, we assumed a 4‐year generation time for lake whitefish.

### 2.7 Adaptive Variation

Evidence of adaptive variation among collections in Lake Erie was evaluated using the WGS dataset by comparing individuals from Long Point (East Basin), Maumee Bay, and the Niagara and Toussaint reefs (West Basin) to identify regions of the genome with elevated differentiation beyond background genetic drift. Evidence of adaptive variation between Lake Huron and Lake Erie was evaluated by comparing the 15 individuals sampled in Coreyon Reef, Lake Huron, to the 38 individuals from Niagara and Toussaint reefs. Two methods were used to identify putative regions of divergence, and then further analysis was focused on only the regions identified by both approaches. First, pairwise windowed *F*_ST_ scans were calculated among all sampling groups using SNPr with a window size of 200 kb and a step of 50 kb. This window size was selected after preliminary assessments showed that it generally provided windows containing 10–30 SNPs, which was decided to be sufficient for accurate estimates of regional *F*_ST_. Based on a genome length 2.8 Gbp and approximately 2800 centimorgans (cM), this should represent about a 0.2 cM window (Mérot et al. 2023; Rogers et al. 2007). The smoothed *F*_ST_ score for each window was converted to a *Z*‐score based on the average *F*_ST_ calculated for all windows on a given chromosome. Windows exceeding a *Z*‐score of 6 with a minimum of 10 SNPs were considered putative adaptive regions. Second, cross‐population extended haplotype homozygosity (XP‐EHH; Szpiech 2024). The XP‐EHH analysis was conducted only on three pairwise comparisons based on early assessments of population structure: Coreyon Reef, Lake Huron versus West Basin reefs (Niagara and Toussaint), Maumee Bay versus West Basin reefs, and Long Point versus West Basin reefs. XP‐EHH uses phased‐haplotype information to identify alleles that have risen to near fixation in one, but not all populations, indicative of a soft or hard sweep. As required by *selscan*, prior to XP‐EHH analysis, data for each chromosome were phased and imputed using Beagle (Browning et al. 2018; Browning and Browning 2007). An XP‐EHH score was calculated using *selscan* v.2.0 for each locus whereby strongly positive scores indicate loci with evidence of a selective sweep in population “A” while strongly negative scores indicate evidence of a selective sweep in population “B.” These scores are calculated for each locus using a window of 100 kb extending in either direction of the target locus (200 kb window). Scores were then normalized following the approach outlined in Szpiech et al. (2021). Using the *norm* module of *selscan*, we computed normalized XP‐EHH scores for each locus, then used a custom R script to identify the proportion of extreme normalized XP‐EHH scores > 2 or < −2 within 100 kb non‐overlapping windows on each chromosome. This window size was selected to match post‐processing scripts and analyses discussed in Rahman et al. (2025, 2026). Windows from all chromosomes containing more than 10 SNPs were then grouped into 10 quantile bins, and the top 1% of scores in each bin were retained as putatively under selection. The intersection of *ZF*_ST_ outlier windows and XP‐EHH windows was then identified using the GenomicRanges R package (Lawrence et al. 2013). The windows identified by both approaches were retained as putative islands of divergence, also known as outlier regions. The association of the regions identified in the complete dataset with spatial structure across all samples was evaluated by filtering the SNP dataset to retain only SNPs in the outlier regions and then conducting PCA using all individuals from Coreyon Reef, Lake Huron, and Lake Erie. For regions identified in only Lake Erie, SNPs were again filtered to retain only SNPs within these outlier regions, and then a PCA was conducted using only individuals from Lake Erie. By conducting this analysis hierarchically and removing Lake Huron samples, associations of regions expressing lower levels of genetic differentiation within Lake Erie were able to be evaluated.

## 3 Results

### 3.1 Sampling

Lake whitefish were successfully collected from the Detroit River and all three basins of Lake Erie (Table 1). Catch effort was impossible to estimate for those captured by commercial fishing operations (e.g., Maumee Bay, Ohio, and Long Point Bay, Ontario), but evidence of elevated harvest of lake whitefish in both areas compared to other times of the year indicates that adult lake whitefish were aggregating in these areas leading up to the spawning season. In areas intentionally targeted by USGS fisheries scientists, effort is more quantifiable at a coarse level. Sample size targets were reached in only a few overnight sets at Niagara Reef and Toussaint Reef in the West Basin. However, extensive effort was required and yielded few fish in the Detroit River and Fairport Harbor regions, suggesting that the relative densities in these areas may be low. Some fish were captured via boat electrofishing during an acoustic telemetry fish tagging operation inside the Detroit River near Fighting Island by OMNR scientists. A total of *N* = 371 lake whitefish were captured in total, including lake whitefish from the Detroit River, the West Basin, the Central Basin, and the East Basin of Lake Erie (Figure 1).

### 3.2 GT‐Seq

Of the 371 captured lake whitefish, DNA was successfully extracted from 340 individuals and sequenced using the Lake Michigan GT‐seq panel. Of these 340 fish, 331 individuals were successfully genotyped to at least an 80% genotyping rate and retained for analysis. Genotype quality control was conducted across these 331 individuals, which retained 343 loci that passed the QC filters and had at least two alleles and < 20% missing genotypes (Table S3). Principal component analysis indicated clear differences in allele frequency between Coreyon Reef in Lake Huron and all of Lake Erie (Figure 2A) and no subgrouping within Lake Erie once Coreyon Reef was excluded (Figure 2B). These results were supported by Pairwise *F*_ST_ analysis, which indicated the highest Pairwise *F*_ST_ between Coreyon Reef and all the Lake Erie spawning sites and low Pairwise *F*_ST_ among spawning sites in Lake Erie (Table 2).

**FIGURE 2:**
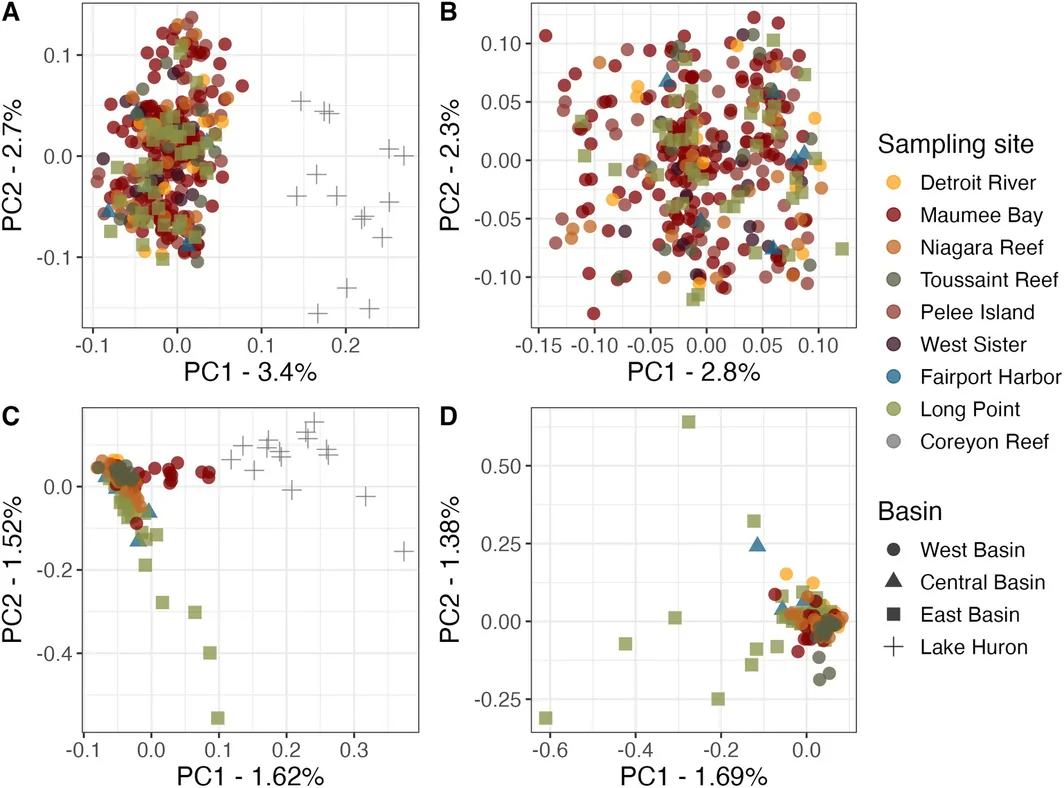
Principal component biplots showing the first two axes of variance for GT‐seq (A, B) and whole‐genome sequencing (C, D) data sets. PCAs were run twice for each dataset, once using all individuals (A, C) and once using only individuals sampled within Lake Erie (B, D).

**TABLE 2:** Pairwise Weir and Cockerham *F*_ST_ estimates from GT‐seq data (located above the diagonal), and estimates made from linkage‐disequilibrium filtered whole‐genome sequencing (below the diagonal). NA values indicate locations genotyped with GT‐seq that were not included in the whole‐genome sequencing assessment. Coreyon Reef is located in Saginaw Bay, Lake Huron, and all other sites are in Lake Erie.

	Coreyon Reef	Detroit River	Maumee Bay	Niagara Reef	Pelee Island	Toussaint Reef	West Sister Is.	Fairport Harbor	Long Point
Coreyon Reef		0.111	0.105	0.108	0.114	0.105	0.118	0.106	0.107
Detroit River	0.017		0.009	0.002	0.013	0.007	0.009	0.001	0.006
Maumee Bay	0.013	0.004		0.003	0.004	0.004	0.007	0.008	0.005
Niagara Reef	0.018	0.003	0.003		0.01	0.001	0.005	−0.003	0.001
Pelee Island	NA	NA	NA	NA		0.008	−0.002	0.015	0.011
Toussaint Reef	0.019	0.005	0.005	0.003	NA		0.016	0.008	0.004
West Sister Is.	NA	NA	NA	NA	NA			0.003	0.014
Fairport Harbor	0.019	0.004	0.007	0.004	NA	0.008	NA		0.004
Long Point	0.016	0.001	0.002	0.002	NA	0.002	NA	0.003	

### 3.3 WGS

All 127 individuals were sequenced successfully, and final genotypes had an average sequencing depth of coverage of 14.4×, with generally < 10% missing data per individual, and limited amounts of variants deviating from expected patterns of Hardy–Weinberg equilibrium and allele frequency distributions (Figure S2). Once all QC filters were applied, we retained 5,444,182 SNPs for the total dataset being used for demographic history and adaptive variation analyses, and 662,623 SNPs in the linkage disequilibrium‐filtered dataset being used for the population structure analysis.

### 3.4 Population Structure

Population structure observed using the WGS data largely reflected GT‐seq results. Principal component analysis using all SNPs indicated clear differentiation between Coreyon Reef in Lake Huron and all of Lake Erie (Figure 2C) and no subgrouping within Lake Erie once Coreyon Reef was excluded (Figure 2D). One difference, however, was an apparent differentiation between lake whitefish from Maumee Bay and the rest of the fish from Lake Erie when all sample locations were included. However, this differentiation was no longer clear once fish from Coreyon Reef were removed. Additionally, pairwise *F*_ST_ was consistent with GT‐seq results, indicating low pairwise *F*_ST_ among all collections in Lake Erie, including between Maumee Bay and the other Lake Erie collections (Table 2).

### 3.5 Demographic History

To explore the potential genetic impacts of historic lake whitefish population declines, the effective population size for the last 150 generations was investigated separately for Lake Erie and Lake Huron. Because lake whitefish mature between 3 and 5 years of age, this accounts for approximately the last 300–750 years (van Oosten and Hile 1949) and approximately measures changes in population since European colonization of the region. Breakpoint analysis, which identified the approximate inflection point after which the trend changes, indicated that there were three changes in the effective population size in Lake Erie. There was an increase in the average effective population size in Lake Erie that began 113–115 generations before present to around 10,000 individuals, and then a second, slower increase to around 16,000 individuals that began around 75–77 generations before present. There was a final change point that began 21–27 generations before present, after which the effective population size went through a 4.3‐fold decline to only around 3700 individuals over the course of 10–20 generations, which coincides with known declines in the lake whitefish fishery harvest rates (Figure 3; Figure S3). Estimates of effective population size were much more variable for Coreyon Reef, Lake Huron, likely due to the much smaller sample size of available individuals (*N* = 15). Only one breakpoint was identified between 56 and 59 generations before present. However, visually, the Lake Huron population appeared to go through a similar increase in effective population size 80–100 generations before present, followed by a more recent decline 5–20 generations before present (Figure 3). Overall, the demographic trajectory of both Huron and Erie populations was similar, indicating a recent decline in effective population size.

**FIGURE 3:**
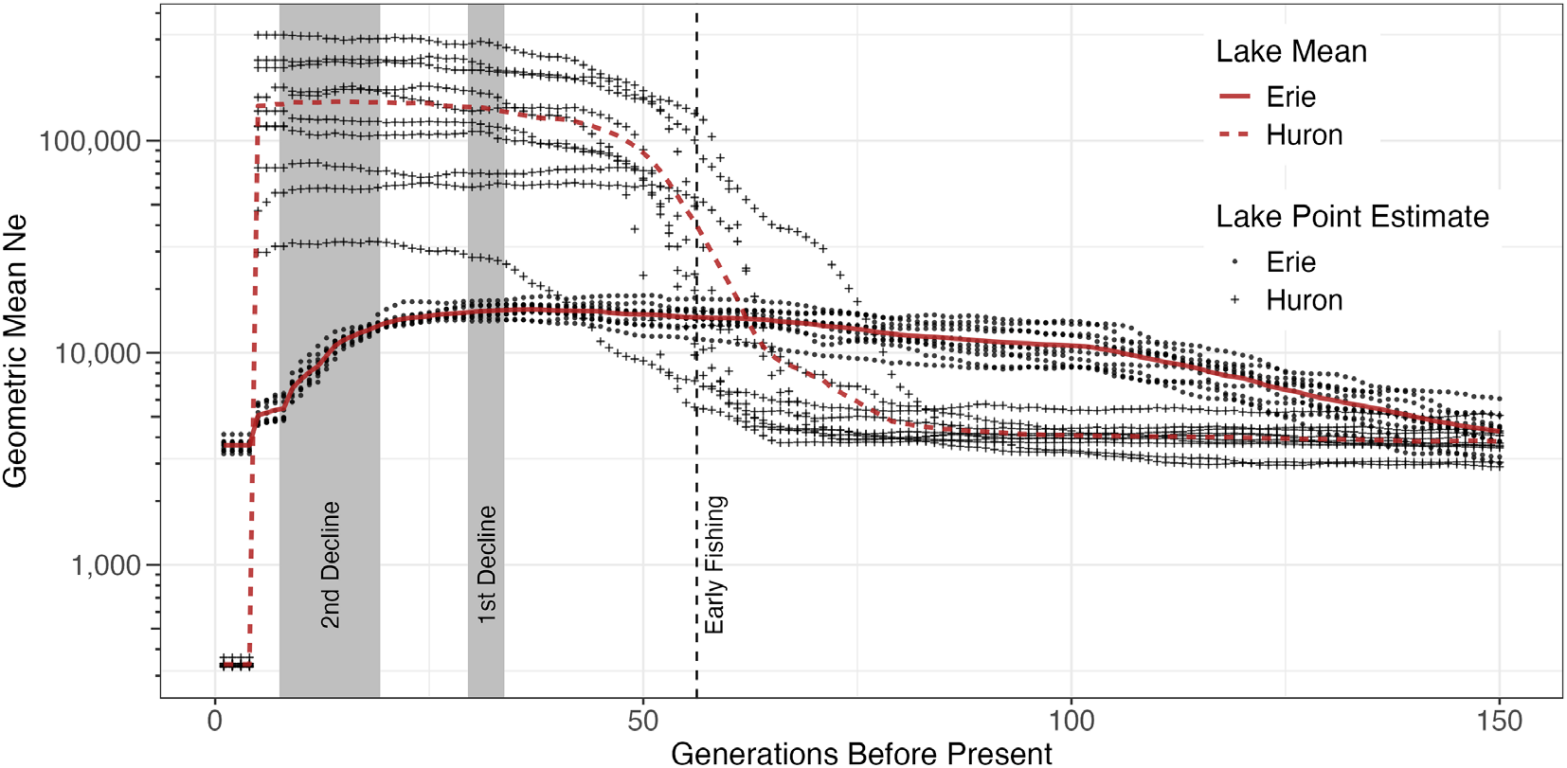
Projected demographic history (effective population size) estimated from 112 lake whitefish from Lake Erie (dots) and 15 Lake Huron (crosses) for the most recent 150 generations, or approximately 450–750 years, assuming a 3‐ to 5‐year generation time. The analysis was run 10 times for each lake to quantify variation in estimates driven by genotyped loci using random subsets of 500,000 loci. Individual replicates are shown in black, and a rolling average is shown in red. The present day is indicated as generation 0 on the *X*‐axis. Note that the *Y*‐axis is log‐10 normalized. The vertical dotted line indicates the approximate generations before present when early fishing by European colonizers would have taken place (year 1800). The gray bars represent the periods of time when intense harvest led to subsequent declines in harvest rates (years 1890–1907 and 1948–1995). These periods were estimates based on historical records of catch and descriptions of the fishery (Regier et al. 1969). Generations before present were estimated by dividing the number of years before present by an approximate time to maturity of 4 years for lake whitefish.

### 3.6 Adaptive Variation

Several genomic islands of differentiation were identified between Lake Erie populations and Coreyon Reef collections in Lake Huron (Figure 4). Regions of high *F*_ST_ were distributed across the genome without any conspicuously large islands that would have been indicative of strong selective sweeps for a particular locus. *ZF*_ST_ analysis identified 166 windows with relatively high *F*_ST_ scores between Coreyon Reef and either Toussaint or Niagara Reef. Of these, 19 overlapped with putative selective sweep regions identified using XP‐HMM. These 19 regions represent potential portions of the genome under recent selection and had an average pairwise *F*_ST_ of 0.174. A principal component analysis using the 1198 SNPs in these outlier regions discriminated Coreyon Reef from the rest of Lake Erie along PC1 and accounted for 29.4% of the genetic variation explained. PC2 explained an additional 14% of the variation, but the PC2 variation did not appear to correspond to spatial structure.

**FIGURE 4:**
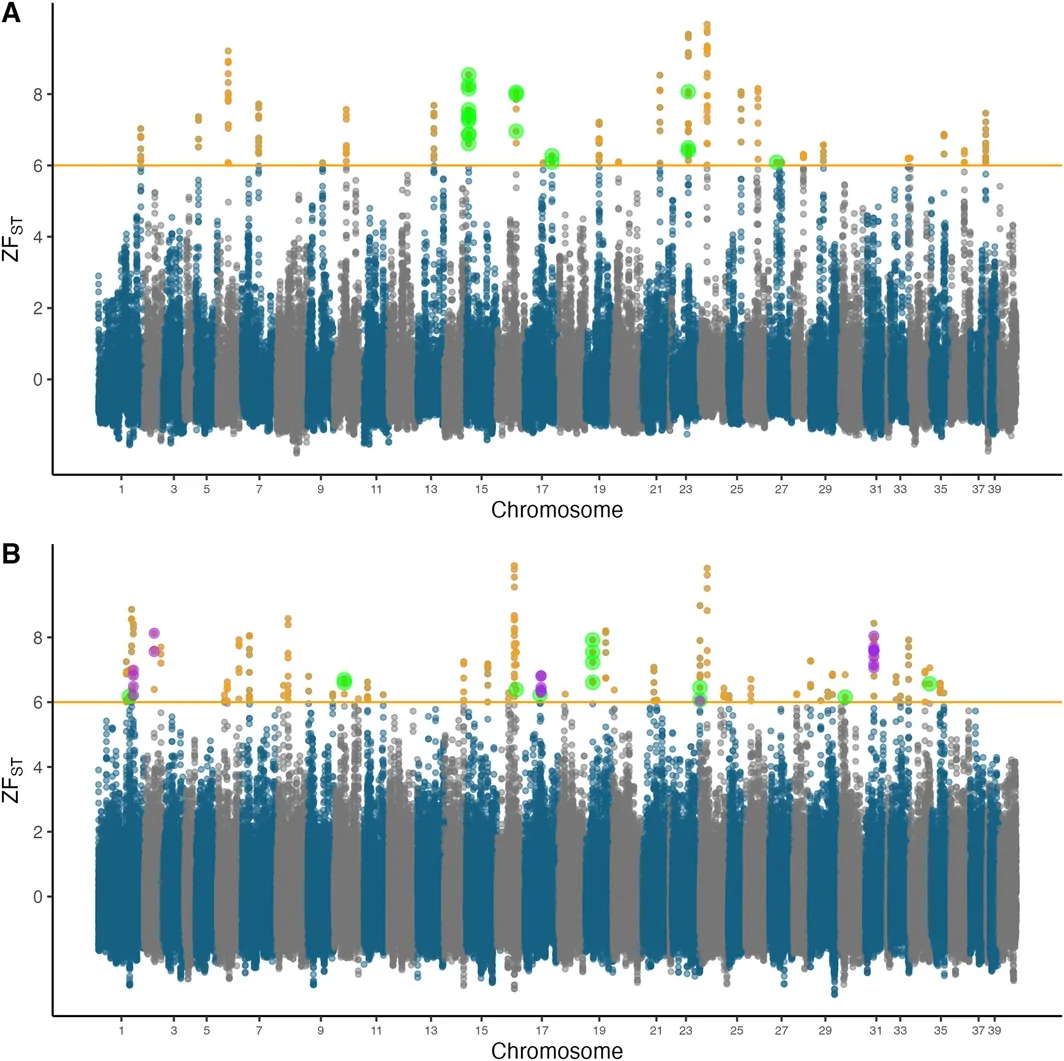
Manhattan plots showing pairwise *ZF*_ST_ scores of 200 kb windows of between (A) Coreyon Reef, Lake Huron, and Niagara and Toussaint reefs, Lake Erie, and between (B) Maumee Bay and Niagara and Toussaint reefs, and Long Point and Niagara and Toussaint reefs in Lake Erie. Loci defined as *ZF*_ST_ outliers have *Z*‐scores > 6 and are shown in yellow and above the yellow horizontal line. Significant windows identified with both *ZF*_ST_ and XP‐HMM are shown in green and purple. Green points show significant windows isolating Coreyon Reef, Lake Huron, and Niagara and Toussaint reefs in (A) and significant windows separating Long Point and Niagara and Toussaint reefs in (B). Purple points show significant windows separating Maumee Bay and Niagara and Toussaint reefs in (B).

When samples from Lake Huron were removed and only within Lake Erie, pairwise *F*_ST_ was investigated; much lower pairwise *F*_ST_ was estimated among all of the collections compared (Maumee Bay, Long Point, and Toussaint and Niagara reefs). Regions of high *F*_ST_ were distributed across the genome with no conspicuously large islands indicative of strong selective sweeps for a particular locus. However, 66 *ZF*_ST_ windows isolated Maumee Bay from the reef collections, and 73 *ZF*_ST_ windows distinguished Long Point from the reef collections. Of the 66 windows isolating Maumee Bay from Niagara and Toussaint reefs, 20 overlapped with putative selective sweep regions identified with XP‐EHH. These 20 regions contained an average *F*_ST_ of 0.066. Of the 73 windows isolating Long Point from Niagara and Toussaint reefs, 13 overlapped with putative selective sweep regions identified with XP‐EHH. These 13 regions contained an average *F*_ST_ of 0.041. A principal component analysis using the 3867 SNPs contained in the outlier regions partially discriminated Niagara and Toussaint reefs from Maumee Bay along PC1 and PC2 and accounted for 8.1% and 7.9% of the variation explained, respectively. However, groupings of individuals were still overlapping in principal component space (Figure S4). The East Basin site, Long Point, was partially discriminated from the rest of Lake Erie along PC3 and PC4 and accounted for 6.7% and 5.5% of the variation explained, respectively. However, these groupings were also highly overlapping in principal component space.

## 4 Discussion

Our results show how system‐specific patterns of stock structure and gene flow can differ throughout a species range that may be influenced by population declines. Lake whitefish show clear and distinguishable spawning stocks in Lake Michigan and Huron (Shi et al. 2022), as well as other large lakes such as Great Slave Lake (Hénault et al. 2026) and, to a lesser degree, Lake Champlain (Euclide et al. 2019). Furthermore, European lake whitefish *C. lavaretus* show a moderate degree of population substructure in the Baltic and North Sea around Finland (Tengstedt et al. 2025). However, despite its size and extensive spawning habitat, Lake Erie appears to contain a nearly panmictic population of lake whitefish. Management decisions regarding lake whitefish in Lake Erie are often based on the presumption that there is a single stock spawning in the West Basin of the lake (Ebener et al. 2021; Stott et al. 2013). Our results support that there is very little distinguishable genetic substructure within Lake Erie, despite indicating that spawning may happen more broadly than in just the West Basin. This contrasts with Euclide et al. (2022), who suggested minor genetic structure might be present based on 88 lake whitefish, but is consistent with other studies like Stott et al. (2013). Our collection efforts yielded many lake whitefish in spawning condition around Long Point in the East Basin of the lake, suggesting there is some spawning occurring beyond the West Basin, which corroborates with other studies (Amidon et al. 2024; Oldenburg et al. 2007; Schaefer et al. 2021). Moderately high spawning site fidelity has been observed in Lake Michigan (Izzo et al. 2024). In Lake Erie, acoustic telemetry studies have indicated moderate to high levels of spawning site fidelity for lake whitefish on West Basin reefs (Kraus et al. 2024). Furthermore, moderate levels of site fidelity were enough to maintain genetic isolation between Lake Erie's East and West Basins for walleye populations that often share spawning sites with lake whitefish (e.g., Euclide et al. 2021; Stepien and Faber 1998). This suggests that dispersal is likely at least partially limited among spawning sites. Therefore, the mechanism driving stock structure in these other systems must differ from Lake Erie in some way.

One possible explanation for the lack of population structure is that lake whitefish were genetically structured in Lake Erie in the past, but large population declines associated with habitat destruction and overfishing led to local extinctions, increased gene flow, and straying during recent generations, resulting in panmictic genetic structure. Like the increased hybridization and loss of ecotype diversity in Lake Constance in European lake whitefish (Haase et al. 2025). Fishing pressure on lake whitefish in Lake Erie was so intense that they were twice fished out of economic viability (Langlois 1954; Oldenburg et al. 2007). Overharvest may have led to an Allee Effect whereby small subpopulations, like those suggested to exist outside of the West Basin, were driven to or near local extinction due to mate limitations (Kramer et al. 2009; Wittmann et al. 2014). Without historical samples, it is difficult to conclusively prove local extinctions occurred. However, small stocks can be accidentally overharvested without proper mixed‐stock management (e.g., Connors et al. 2019; Isermann et al. 2020; Ying et al. 2011). Observations of spawning in the Detroit River (Roseman et al. 2007; Stott et al. 2024) and Fairport Harbor are all recent, within the last two decades, suggesting that these sites could have been completely extirpated in the past. Assuming local extinctions occurred, spawning sites would then have been most likely recolonized by West Basin lake whitefish, resulting in a rapid homogenization of population structure lake‐wide (Bett et al. 2017). A similar pattern of reduced population structure of lake trout has also been hypothesized to have occurred in Lake Superior following large declines in abundance (Baillie et al. 2016) and in European alpine whitefish species because of eutrophication (Vonlanthen et al. 2012; Alexander et al. 2017). Therefore, it is not unreasonable to conclude that loss of abundance can lead to a loss of genetic differentiation.

Stocking fish from hatcheries can also substantially impact population structure and genetic diversity (Duftner et al. 2005; Marie et al. 2010). During the same period that lake whitefish abundance was declining, multiple hatcheries were established in the West Basin of Lake Erie to bolster population abundance that likely sourced their brood stock from the West Basin (Langlois 1954). Supplemental hatchery stocking has been demonstrated to limit genetic diversity and can directly harm wild populations through hybridization, introgression, and reduced fitness (Araki and Schmid 2010). When combined with widespread population decline, the addition of hatchery fish introgression with wild fish could have disrupted genetic structure, contributing to panmixia.

Immigration following overharvest can rapidly add new alleles to depleted populations and expedite recovery. Simulations in Atlantic cod *Gadus morhua* suggested that population recovery following an Allee Effect took between 20 and 100 years when immigration was 5%–7.5% (Kuparinen and Uusi‐Heikkilä 2020). If immigration did neutralize population structure, this could explain why we did not identify any substantial islands of genetic divergence that would indicate there are locally adaptive alleles associated with certain spawning populations (Bierne et al. 2013; Bradbury et al. 2013). Instead, we identified multiple regions with moderately high *F*_ST_ distributed across the genome, which is more consistent with the early stages of genetic drift or selection across many loci that are only loosely associated with geography or spawning site. While several regions were found that appeared to contain outlier loci and evidence of a selective sweep, further inspection of these regions found inconsistent grouping of individuals via PCA, indicating that these regions may be false positive detections or detections of regions under selection more broadly that do not closely correspond to spawning sites (Bierne et al. 2013). Our results do not mean that selection and local adaptation are not ongoing in Lake Erie or Lake Huron but suggest that the strength of directional selection associated with spawning site geography is not great enough to overcome genetic drift or is in very early stages of divergence. This finding is therefore more consistent with recolonization adding alleles from neighboring populations (Kuparinen and Uusi‐Heikkilä 2020) and possibly early stages of local adaptations like those shown by invasive species experiencing the Allee Effect along range expansions (e.g., Lachmuth et al. 2011). Future work incorporating archival samples from before lake whitefish population declines could provide evidence of whether locally adapted lake whitefish stocks existed in Lake Erie.

Overharvest and anthropogenically induced bottlenecks, and climatic events can have substantial, if variable, impacts on effective population size (Sadler et al. 2023). Bluefin tuna show signs of demographic decline associated with harvest (Andrews et al. 2025), and Atlantic herring have been suggested to have had stock‐specific reactions to harvest and the Little Ice Age (Atmore et al. 2022). We found similar patterns in Lake whitefish in both Lake Erie and Lake Huron, which maintained relatively large effective population sizes for most of the last 100 generations, but showed major transition points that corresponded to harvest and climate. First, there was a potential population expansion approximately 100 generations before present, possibly corresponding to the end of the Little Ice Age that took place between 1570 and 1900 (Matthews and Briffa 2005). Second, there was a substantial drop in effective population size around the time of the last known major decline in the lake whitefish fishery. This hump‐like pattern in effective population size can appear when metapopulation dynamics are not completely accounted for (Santiago et al. 2025). The evidence of panmixia was clear in Lake Erie. However, it is possible that this may explain the pattern observed in Lake Huron, where our sample size was small and the rise and ultimate fall of effective population size were much more variable. Therefore, additional samples from Lake Huron would be needed to conclusively measure the demographic history in this lake. Nonetheless, there are legitimate ecological reasons for a major decline in effective population size in both lakes. The recent 4.3‐fold decline in effective population size in Lake Erie, corresponding with a similar decline in Lake Huron, is consistent with a genetic bottleneck related to declines in lake whitefish fishery productivity (Figure S3). Lake whitefish throughout the Great Lakes experienced major declines over the last 100 years (Ebener et al. 2021). These declines were in part due to commercial fishing, but this period of time also corresponds with other substantial stressors, such as a high abundance of invasive sea lamprey and substantially degraded habitat and water quality throughout the Great Lakes (McKenna 2019). Abundance declined substantially in the 1890s and the late 1950s, leading to the de‐commercialization of the lake whitefish fishery (Langlois 1954; Oldenburg et al. 2007). Genetic bottlenecks often require substantial and elongated declines in population size to reduce heterozygosity and purge low‐frequency alleles from a population (Nei et al. 1975). Once no longer profitable, harvesters stopped targeting lake whitefish as intensely. This, combined with the size of Lake Erie and the large amount of suitable spawning habitat, means that recruitment after these declines likely remained moderately high, and may have been enough to maintain much of the genetic diversity held within the system. This may explain why the effective population size is still in the thousands in Lake Erie. Similarly, lake trout and bloater *Coregonus hoyi* were nearly extirpated from the lower Great Lakes during this same time and show only a modest decrease in allelic richness (Favé and Turgeon 2008; Guinand et al. 2003). The loss of genetic diversity of lake whitefish suggests that they have experienced enough of a population decline to significantly bottleneck genetic diversity. Overall effective population sizes and heterozygosity remain moderately high, meaning that there is likely adaptive potential still held within standing genetic variation (García‐Dorado and Caballero 2021; Seaborn et al. 2021). Nonetheless, the genetic diversity lost during these declines may have implications for the adaptive capacity of lake whitefish today that may change how they are able to respond to rapid environmental changes via standing genetic diversity (Chaturvedi et al. 2021; Embke et al. 2026).

One important methodological conclusion of our study is that patterns of genetic structure and diversity were nearly identical between low‐density GT‐seq data and high‐density whole‐genome sequencing data. This indicates, first, that ascertainment bias likely did not have a substantial impact on conclusions made by GT‐seq data (Bradbury et al. 2011). This is an important consideration in GT‐seq panel development and application (Lachance and Tishkoff 2013), and our study suggests that a broader application of the panel within the Great Lakes region may be possible. Second, while due to the panmixia in Lake Erie, genetic stock identification within the lake is not feasible, distinguishing between Lake Huron and Lake Erie was, which could be useful for identifying migration events or inter‐lake mixed‐stock harvest. While inter‐lake movement is not well established for lake whitefish, they do have the capacity for long movements (Ebener et al. 2010; Kraus et al. 2024). Inter‐lake movements by fish are not uncommon in the Great Lakes and have been well documented in several species, including walleye, lake sturgeon *Acipenser fulvescens* , and muskellunge *Esox masquinongy* (Fissette et al. 2025; Hessenauer et al. 2021; Brenden et al. 2015). We also noted higher estimates of genetic diversity using the GT‐seq panel compared to the whole‐genome survey. This is because the loci in the GT‐seq panel were specifically chosen to maximize the panel's utility for population genetic and kinship studies, which require higher levels of minor allele frequency and heterozygosity than might be observed across a full genome survey (Dussault and Boulding 2018; Chang et al. 2021). Therefore, loci with low minor allele frequency were filtered out during development (Krause 2025). The GT‐seq panel is a consistent set of loci that are already being evaluated throughout the Great Lakes. Therefore, future studies that are interested in comparing genetic diversity within the Great Lakes should consider using this same GT‐seq panel for their assessment. If a randomly reduced representation approach, like RADseq or genotyping by sequencing, is used to genotype loci, comparing their results to our genome‐wide estimates would provide a more accurate comparison (Andrews et al. 2016). While the GT‐seq panel may not provide a means to directly conduct stock assignment in Lake Erie, genotypes obtained from this approach will still provide useful information about genetic diversity, kinship, and inter‐lake movements within Lake Erie and surrounding systems.

## 5 Conclusions

Population declines can have substantial and complex impacts on genetic diversity in population genetic structure. In many cases, these declines can result in substantial loss of genetic diversity, while others do not (Sadler et al. 2023). In the Great Lakes, multiple fish species have experienced substantial population‐level fluctuations due to a combination of anthropogenic impacts and intensive management, which has altered population, demography, and genetic structure (Favé and Turgeon 2008; Guinand et al. 2003; Euclide et al. 2024). Our study indicates that lake whitefish in Lake Erie also underwent a substantial demographic decline that permanently impacted effective population size and may have coincided with a loss of significant stock structure within the lake. Despite lake‐wide spatial coverage of our lake whitefish spawning aggregation collections, lake whitefish in Lake Erie are not genetically structured, and there is no strong evidence of locally adaptive loci differentiating putative spawning aggregations. This is in direct contrast to what is seen in other similar large‐lake populations in Lake Michigan and Great Slave Lake (Shi et al. 2022; Hénault et al. 2026). Based on the large demographic decline, we hypothesize that Lake Erie may have had a structured population in the past, but additional studies using archival samples would be needed to test this. Other methods that quantify contemporary spatial structure through behavior, such as acoustic telemetry, would also be helpful to determine whether there are ecologically distinct spatial groups of lake whitefish in Lake Erie that are unable to be detected using genetic data (Hawkins et al. 2016; Waples 1998; Waples and Gaggiotti 2006). Presently, our research supports ongoing management directives that presume there is only a single stock of lake whitefish. Furthermore, these results provide a sentinel study highlighting how genetic diversity can rapidly change in the face of anthropogenic stressors. Lake whitefish and other species that have supported commercial and recreational fisheries in the Great Lakes have likely experienced the most direct population impacts within the last 100 years. This study therefore adds to the growing body of literature that identifies the Great Lakes region as a case study for climate and environmental disturbance (Collingsworth et al. 2017; McKenna 2019). Results like ours support the idea that adaptive capacity and genetic diversity may have been impacted in many other species that are less studied and lack long‐term datasets of population abundance. Therefore, these results provide beneficial knowledge that can be considered when working toward species restoration and conservation goals.

## Funding

This work was supported by the U.S. Fish and Wildlife Service through the Great Lakes Fish and Wildlife Restoration Act (GLFWRA) [F22AP02898].

## Conflicts of Interest

The authors declare no conflicts of interest.

## Supporting information


**Figure S1:** Exact collection locations for *N* = 371 Lake Whitefish collected for the present study. Genetic samples were collected from Lake Whitefish in the Detroit River and the west, central, and east basins of Lake Erie.
**Figure S2:** Summary of whole‐genome sequencing dataset containing 127 lake whitefish and 5,444,182 retained SNP loci including (A) percent missing data by average depth of coverage (DOC) for each individual, (B) distribution of locus average DOC across all samples where the red line indicates the mean DOC, (C) distribution of Hardy–Weinberg equilibrium one‐tailed test for heterozygosity excess, and (C) final distribution of allele frequencies of the reference allele (black) and alternate allele (red).
**Figure S3:** Commercial harvest data for Lake Whitefish in Lake Erie spanning 1867–2020. Commercial harvest of Lake Whitefish crashed in the late 1950s after reaching an annual high of just over 7 million lbs.
**Figure S4:** Principal component analysis (PCA) biplots of the first to axes using only SNPs found within outlier windows detected among Lake Huron and Niagara and Toussaint Reefs (A, B), and among Lake Erie spawning site collections (C, D).

**Table S1:** While sampling efforts at known Lake Whitefish spawning aggregations (e.g., Niagara Reef, Maumee Bay) produced significant catches, exploratory efforts in the central basin of Lake Erie (e.g., Fairport Harbor) and the Detroit River resulted in minimal catches despite significant efforts during the fall of 2022 and 2023.

**Table S2:** Filtering parameters and steps used to obtain the final SNP dataset used for whole‐genome sequencing. Filtering occurred in a stepwise fasion, programs, flags, and a general description of each step is included.

**Table S3:** Per‐locus diversity statistics broken down by collection. Minor allele frequency (maf), major and minor allele counts (maj.count, min.count) and alleles (major, minor), nucleotide diversity (pi), observed and expected heterozygosity (ho, he), corrected private allele count (pa_corrected), and inbreeding coefficent (fis).

## Data Availability

All sample metadata files can be found via Geome‐db (https://n2t.net/ark:/21547/R2649), and whole‐genome sequencing files can be found via the Sequence Read Archive on NCBI following the publication of these data PRJNA1402938. GT‐seq data is available upon request from the corresponding author or via IUDataCore: https://doi.org/10.5967/3e14‐xg27. Associated code for the project can be found on GitHub: https://github.com/peuclide/GenBottleAndPanmixia_ErieLWF.

## References

[eva70337-bib-0001] Alexander, T. J. , P. Vonlanthen , and O. Seehausen . 2017. “Does Eutrophication‐Driven Evolution Change Aquatic Ecosystems?” Philosophical Transactions of the Royal Society of London, Series B: Biological Sciences 372, no. 1712: 20160041. 10.1098/RSTB.2016.0041. PMC5182437 27920386

[eva70337-bib-0002] Allan, J. D. , R. Abell , Z. Hogan , et al. 2005. “Overfishing of Inland Waters.” Bioscience 55, no. 12: 1041. 10.1641/0006-3568(2005)055[1041:OOIW]2.0.CO;2.

[eva70337-bib-0003] Amidon, Z. J. , R. L. DeBruyne , E. F. Roseman , and C. M. Mayer . 2021a. “Spatial Extent of Contemporary Lake Whitefish Spawning in Western Lake Erie.” Advances in Limnology 66: 163–172. 10.1127/ADV_LIMNOL/2021/0063.

[eva70337-bib-0004] Amidon, Z. J. , R. L. DeBruyne , E. F. Roseman , and C. M. Mayer . 2021b. “Contemporary and Historic Dynamics of Lake Whitefish ( *Coregonus clupeaformis* ) Eggs, Larvae, and Juveniles Suggest Recruitment Bottleneck During First Growing Season.” Annales Zoologici Fennici 58, no. 1–3: 161–175. 10.5735/086.058.0405.

[eva70337-bib-0005] Amidon, Z. J. , R. L. DeBruyne , E. F. Roseman , C. M. Mayer , and A. Sakas . 2024. “Larval Lake Whitefish ( *Coregonus clupeaformis* ) Zooplankton Consumption Remains Constant Despite Variation in Prey Densities in Western Lake Erie.” Aquatic Ecosystem Health & Management 27, no. 1: 85–97. 10.14321/AEHM.027.01.85.

[eva70337-bib-0006] Andrews, A. J. , E. F. Eriksen , B. Star , et al. 2025. “Ancient DNA Suggests a Historical Demographic Decline and Genetic Erosion in the Atlantic Bluefin Tuna.” Proceedings of the National Academy of Sciences of the United States of America 122, no. 21: e2409302122. 10.1073/pnas.2409302122 PMC12130816 40392844

[eva70337-bib-0007] Andrews, K. R. , J. M. Good , M. R. Miller , G. Luikart , and P. A. Hohenlohe . 2016. “Harnessing the Power of RADseq for Ecological and Evolutionary Genomics.” Nature Reviews Genetics 2, no. 17: 81–92. 10.1038/nrg.2015.28. PMC4823021 26729255

[eva70337-bib-0008] Araki, H. , and C. Schmid . 2010. “Is Hatchery Stocking a Help or Harm?: Evidence, Limitations and Future Directions in Ecological and Genetic Surveys.” Aquaculture 308: S2–S11. 10.1016/j.aquaculture.2010.05.036.

[eva70337-bib-0009] Atmore, L. M. , L. Mart Inez‐Garc Ia , D. Makowiecki , et al. 2022. “Population Dynamics of Baltic Herring Since the Viking Age Revealed by Ancient DNA and Genomics.” Proceedings of the National Academy of Sciences of the United States of America 119, no. 45: e2208703119. 10.1073/pnas.2208703119. PMC9659336 36282902

[eva70337-bib-0010] Baillie, S. M. , A. M. Muir , K. Scribner , P. Bentzen , and C. C. Krueger . 2016. “Loss of Genetic Diversity and Reduction of Genetic Distance Among Lake Trout *Salvelinus namaycush* Ecomorphs, Lake Superior 1959 to 2013.” Journal of Great Lakes Research 42, no. 2: 204–216. 10.1016/j.jglr.2016.02.001.

[eva70337-bib-0011] Baldwin, N. A. , R. W. Saalfeld , M. R. Dochoda , H. J. Buettner , R. L. Eshenroder , and R. O'Gorman . 2018. Commercial Fish Production in the Great Lakes 1867–2015. Great Lakes Fishery Commission. http://www.glfc.org/databases/commercial/commerc.php.

[eva70337-bib-0012] Baumsteiger, J. , and J. L. Kerby . 2009. “Effectiveness of Salmon Carcass Tissue for Use in DNA Extraction and Amplification in Conservation Genetic Studies.” North American Journal of Fisheries Management 29, no. 1: 40–49. 10.1577/M07-212.1.

[eva70337-bib-0013] Bett, N. N. , S. G. Hinch , N. J. Burnett , M. R. Donaldson , and S. M. Naman . 2017. “Causes and Consequences of Straying Into Small Populations of Pacific Salmon.” Fisheries 42, no. 4: 220–230. 10.1080/03632415.2017.1276356.

[eva70337-bib-0014] Bierne, N. , D. Roze , and J. J. Welch . 2013. “Pervasive Selection or Is It? Why Are FST Outliers Sometimes So Frequent?” Molecular Ecology 22, no. 8: 2061–2064. 10.1111/mec.12241. 23671920

[eva70337-bib-0015] Blain, S. A. , C. E. Adams , P. A. Amundsen , R. Knudsen , and L. Chavarie . 2025. “Which Ecological Factors Influence the Level of Intraspecific Diversity Within Post‐Glacial Fishes? A Case Study Using *Coregonus* and *Salvelinus* .” Freshwater Biology 70, no. 1: e14357. 10.1111/FWB.14357.

[eva70337-bib-0016] Bolger, A. M. , M. Lohse , and B. Usadel . 2014. “Trimmomatic: A Flexible Trimmer for Illumina Sequence Data.” Bioinformatics 30, no. 15: 2114–2120. 10.1093/bioinformatics/btu170. PMC4103590 24695404

[eva70337-bib-0123] Bootsma, M. L. , K. M. Gruenthal , G. J. McKinney , et al. 2020. “A GT‐seq Panel for Walleye ( Sander vitreus ) Provides Important Insights for Efficient Development and Implementation of Amplicon Panels in Non‐Model Organisms.” Molecular Ecology Resources 20, no. 6: 1706–1722. 10.1111/1755-0998.13226. 32668508

[eva70337-bib-0017] Bradbury, I. R. , S. Hubert , B. Higgins , et al. 2011. “Evaluating SNP Ascertainment Bias and Its Impact on Population Assignment in Atlantic Cod, *Gadus morhua* .” Molecular Ecology Resources 11, no. S1: 218–225. 10.1111/j.1755-0998.2010.02949.x. 21429176

[eva70337-bib-0018] Bradbury, I. R. , S. Hubert , B. Higgins , et al. 2013. “Genomic Islands of Divergence and Their Consequences for the Resolution of Spatial Structure in an Exploited Marine Fish.” Evolutionary Applications 6, no. 3: 450–461. 10.1111/eva.12026. PMC3673473 23745137

[eva70337-bib-0019] Brander, K. 2010. “Impacts of Climate Change on Fisheries.” Journal of Marine Systems 79, no. 3–4: 389–402. 10.1016/j.jmarsys.2008.12.015.

[eva70337-bib-0020] Brenden, T. O. , K. T. Scribner , J. R. Bence , et al. 2015. “Contributions of Lake Erie and Lake St. Clair Walleye Populations to the Saginaw Bay, Lake Huron, Recreational Fishery: Evidence From Genetic Stock Identification.” North American Journal of Fisheries Management 35: 567–577. 10.1080/02755947.2015.1020079.

[eva70337-bib-0021] Browning, B. L. , Y. Zhou , and S. R. Browning . 2018. “A One‐Penny Imputed Genome From Next‐Generation Reference Panels.” American Journal of Human Genetics 103, no. 3: 338–348. 10.1016/j.ajhg.2018.07.015. PMC6128308 30100085

[eva70337-bib-0022] Browning, S. R. , and B. L. Browning . 2007. “Rapid and Accurate Haplotype Phasing and Missing‐Data Inference for Whole‐Genome Association Studies by Use of Localized Haplotype Clustering.” American Journal of Human Genetics 81, no. 5: 1084–1097. 10.1086/521987. PMC2265661 17924348

[eva70337-bib-0023] Chang, S. L. , H. G. M. Ward , and M. A. Russello . 2021. “Genotyping‐in‐Thousands by Sequencing Panel Development and Application to Inform Kokanee Salmon ( *Oncorhynchus nerka* ) Fisheries Management at Multiple Scales.” PLoS One 16: e0261966. 10.1371/JOURNAL.PONE.0261966. PMC8699693 34941943

[eva70337-bib-0024] Chaturvedi, A. , J. Zhou , J. A. M. Raeymaekers , et al. 2021. “Extensive Standing Genetic Variation From a Small Number of Founders Enables Rapid Adaptation in Daphnia.” Nature Communications 12, no. 1: 1–9. 10.1038/s41467-021-24581-z. PMC8280168 34262034

[eva70337-bib-0025] Collingsworth, P. D. , D. B. Bunnell , M. W. Murray , et al. 2017. “Climate Change as a Long‐Term Stressor for the Fisheries of the Laurentian Great Lakes of North America.” Reviews in Fish Biology and Fisheries 27, no. 2: 363–391. 10.1007/s11160-017-9480-3.

[eva70337-bib-0026] Connors, B. , W. Atlas , C. Melymick , M. Moody , J. Moody , and A. Frid . 2019. “Conservation Risk and Uncertainty in Recovery Prospects for a Collapsed and Culturally Important Salmon Population in a Mixed‐Stock Fishery.” Marine and Coastal Fisheries 11, no. 6: 423–436. 10.1002/MCF2.10092.

[eva70337-bib-0027] Danecek, P. , A. Auton , G. Abecasis , et al. 2011. “The Variant Call Format and VCFtools.” Bioinformatics 27, no. 15: 2156–2158. 10.1093/bioinformatics/btr330. PMC3137218 21653522

[eva70337-bib-0028] Duftner, N. , S. Koblmüller , S. Weiss , N. Medgyesy , and C. Sturmbauer . 2005. “The Impact of Stocking on the Genetic Structure of European Grayling ( *Thymallus thymallus* , Salmonidae) in Two Alpine Rivers.” Hydrobiologia 542, no. 1: 121–129. 10.1007/s10750-004-4951-7.

[eva70337-bib-0029] Dussault, F. M. , and E. G. Boulding . 2018. “Effect of Minor Allele Frequency on the Number of Single Nucleotide Polymorphisms Needed for Accurate Parentage Assignment: A Methodology Illustrated Using Atlantic Salmon.” Aquaculture Research 49: 1368–1372. 10.1111/are.13566.

[eva70337-bib-0030] Ebener, M. P. , T. O. Brenden , G. M. Wright , M. L. Jones , and M. Faisal . 2010. “Spatial and Temporal Distributions of Lake Whitefish Spawning Stocks in Northern Lakes Michigan and Huron, 2003–2008.” Journal of Great Lakes Research 36: 38–51. 10.1016/j.jglr.2010.02.002.

[eva70337-bib-0031] Ebener, M. P. , E. S. Dunlop , and A. M. Muir . 2021. Declining Recruitment of Lake Whitefish to Fisheries in the Laurentian Great Lakes: Management Considerations and Research Priorities (Miscellaneous Publication 2021–01). Great Lakes Fishery Commission.

[eva70337-bib-0032] Ebener, M. P. , R. E. Kinnunen , P. J. Schneeberger , L. C. Mohr , J. A. Hoyle , and P. Peeters . 2008. “Management of Commercial Fisheries for Lake Whitefish in the Laurentian Great Lakes of North America.” In International Governance of Fisheries Ecosystems, edited by M. G. Schechter , N. J. Leonard , and W. W. Taylor , 99–143. American Fisheries Society.

[eva70337-bib-0033] Eckmann, R. , and R. Rbsch . 1998. “Lake Constance. Characterization of an Ecosystem in Transition.” Archiv für Hydrobiologie: Special Issues: Advances in Limnology 53: 285–301. http://www.ub.uni‐konstanz.de/kops/volltexte/2007/4007/.

[eva70337-bib-0034] Embke, H. S. , K. M. Alofs , D. O. Bunnell , et al. 2026. “Adaptive Capacity of Freshwater Organisms in North America: Current Understanding and Future Applications.” Global Change Biology Communications 1: e70009. 10.1002/GCB4.70009.

[eva70337-bib-0035] Euclide, P. T. , C. W. Kilpatrick , and J. E. Marsden . 2019. “Genetic Diversity and Structure of Lake Whitefish ( *Coregonus clupeaformis* ) 100 Years After Closure of the Commercial Fishery.” Journal of Great Lakes Research 45, no. 6: 1310–1319. 10.1016/j.jglr.2019.09.010.

[eva70337-bib-0036] Euclide, P. T. , H. Kuhl , C. C. Wilson , et al. 2024. “Human Impacts on Great Lakes Walleye *Sander vitreus* Structure, Diversity and Local Adaptation.” Molecular Ecology 34: e17558. 10.1111/MEC.17558. PMC12684333 39487667

[eva70337-bib-0037] Euclide, P. T. , T. MacDougall , J. M. Robinson , et al. 2021. “Mixed‐Stock Analysis Using Rapture Genotyping to Evaluate Stock‐Specific Exploitation of a Walleye Population Despite Weak Genetic Structure.” Evolutionary Applications 14: 1403–1420. 10.1111/eva.13209. PMC8127713 34025775

[eva70337-bib-0038] Euclide, P. T. , J. D. Schmitt , R. T. Kraus , A. Cook , and J. Markham . 2022. “Genome‐Wide Genetic Diversity May Help Identify Fine‐Scale Genetic Structure Among Lake Whitefish Spawning Groups in Lake Erie.” Journal of Great Lakes Research 48, no. 4: 1083–1090. 10.1016/j.jglr.2022.05.020.

[eva70337-bib-0039] Favé, M. J. , and J. Turgeon . 2008. “Patterns of Genetic Diversity in Great Lakes Bloaters ( *Coregonus hoyi* ) With a View to Future Reintroduction in Lake Ontario.” Conservation Genetics 9, no. 2: 281–293. 10.1007/s10592-007-9339-6.

[eva70337-bib-0040] Fischer, J. L. , J. J. Pritt , E. F. Roseman , et al. 2018. “Lake Sturgeon, Lake Whitefish, and Walleye Egg Deposition Patterns With Response to Fish Spawning Substrate Restoration in the St. Clair–Detroit River System.” Transactions of the American Fisheries Society 147, no. 1: 79–93. 10.1002/tafs.10016.

[eva70337-bib-0041] Fissette, S. D. , C. C. Krueger , L. M. O'Connor , et al. 2025. “Lake Sturgeon Behavioral Diversity in the Laurentian Great Lakes: Migratory Patterns Across Populations and Habitats.” Movement Ecology 13, no. 1: 75. 10.1186/s40462-025-00585-y PMC12548266 41126349

[eva70337-bib-0042] García‐Dorado, A. , and A. Caballero . 2021. “Neutral Genetic Diversity as a Useful Tool for Conservation Biology.” Conservation Genetics 22, no. 4: 541–545. 10.1007/s10592-021-01384-9.

[eva70337-bib-0043] Graham, C. F. , D. R. Boreham , R. G. Manzon , J. Y. Wilson , and C. M. Somers . 2022. “Population Structure of Lake Whitefish ( *Coregonus clupeaformis* ) From the Mississippian Lineage in North America.” Facets 7: 853–874. 10.1139/facets-2021-0191.

[eva70337-bib-0044] Guinand, B. , K. T. Scribner , K. S. Page , and M. K. Burnham‐Curtis . 2003. “Genetic Variation Over Space and Time: Analyses of Extinct and Remnant Lake Trout Populations in the Upper Great Lakes.” Proceedings of the Royal Society of London, Series B: Biological Sciences 270, no. 1513: 425–433. 10.1098/rspb.2002.2250. PMC1691259 12639323

[eva70337-bib-0045] Haase, S. , J. Baer , C. W. Berg , et al. 2025. “New Insights Into the Collapse of Upper Lake Constance Whitefish ( *Coregonus wartmanni* ) and the Impact of Commercial Fisheries Management.” Canadian Journal of Fisheries and Aquatic Sciences 82: 1–10. 10.1139/CJFAS-2024-0370/SUPPL_FILE/CJFAS-2024-0370SUPPLC.DOCX.

[eva70337-bib-0046] Hawkins, S. J. , K. Bohn , D. W. Sims , et al. 2016. “Fisheries Stocks From an Ecological Perspective: Disentangling Ecological Connectivity From Genetic Interchange.” Fisheries Research 179: 333–341. 10.1016/J.FISHRES.2016.01.015.

[eva70337-bib-0047] Hemstrom, W. , and M. Jones . 2023. “snpR: User Friendly Population Genomics for SNP Data Sets With Categorical Metadata.” Molecular Ecology Resources 23, no. 4: 962–973. 10.1111/1755-0998.13721. 36239472

[eva70337-bib-0048] Hénault, P. , R. Bouchard , D. A. Boguski , et al. 2026. “Genome‐Wide Population Structure of Lake Whitefish ( *Coregonus clupeaformis* ) in a Subarctic Great Lake.” Evolutionary Applications 19, no. 6: e70268. 10.1111/EVA.70268. PMC13240244 42253485

[eva70337-bib-0049] Hessenauer, J.‐M. , C. Harris , S. Marklevitz , et al. 2021. “Seasonal Movements of Muskellunge in the St. Clair—Detroit River System: Implications for Multi‐Jurisdictional Fisheries Management.” Journal of Great Lakes Research 47, no. 2: 475–485.

[eva70337-bib-0050] Isermann, D. A. , M. J. Belnap , K. N. Turnquist , et al. 2020. “Defining the Need for Genetic Stock Assignment When Describing Stock Demographics and Dynamics: An Example Using Lake Whitefish in Lake Michigan.” Transactions of the American Fisheries Society 149, no. 4: 398–413. 10.1002/tafs.10235.

[eva70337-bib-0051] Izzo, L. K. , D. J. Dembkowski , T. R. Binder , et al. 2024. “Comparing Conventional Tagging Methods and Acoustic Telemetry to Inform Management of Lake Whitefish in Lake Michigan.” North American Journal of Fisheries Management 44, no. 6: 1232–1248. 10.1002/nafm.11040.

[eva70337-bib-0052] Jombart, T. 2008. “adegenet: A R Package for the Multivariate Analysis of Genetic Markers.” Bioinformatics 24, no. 11: 1403–1405. 10.1093/bioinformatics/btn129. 18397895

[eva70337-bib-0053] Kalejs, N. I. , M. T. Zischke , J. S. Beugly , et al. 2022. “An Evaluation of Fish Spawning on Degraded and Remnant Reefs in Saginaw Bay, Lake Huron.” Journal of Great Lakes Research 48, no. 2: 593–605. 10.1016/j.jglr.2022.01.010.

[eva70337-bib-0054] Kamvar, Z. N. , J. C. Brooks , and N. J. Grünwald . 2015. “Novel R Tools for Analysis of Genome‐Wide SNP Data From Clonal Agricultural Plant Pathogens.” PeerJ 3: e723. 10.7717/peerj.723.

[eva70337-bib-0055] Koenigbauer, S. T. , D. G. Fielder , J. R. Meyer , K. M. Keeler , P. D. Collingsworth , and T. O. Höök . 2026. “A Post‐Restoration Evaluation of Fish Spawning on Coreyon Reef in Saginaw Bay, Lake Huron.” Journal of Great Lakes Research 52: 102819. 10.1016/j.jglr.2026.102819.

[eva70337-bib-0056] Kramer, A. M. , B. Dennis , A. M. Liebhold , and J. M. Drake . 2009. “The Evidence for Allee Effects.” Population Ecology 51, no. 3: 341–354. 10.1007/S10144-009-0152-6.

[eva70337-bib-0057] Kraus, R. T. , H. A. Cook , A. Sakas , et al. 2024. “Risk of Capture Is Modified by Hypoxia and Interjurisdictional Migration of Lake Whitefish ( *Coregonus clupeaformis* ).” Scientific Reports 14: 18061. 10.1038/s41598-024-65147-5. PMC11300607 39103373

[eva70337-bib-0058] Krause, A. 2025. “Spatiotemporal Patterns of Genetic Stock Mixing and Their Application in Mixture‐Informed Catch‐at‐Age Models for Lake Whitefish in Lake Michigan.” Master's thesis, University of Wisconsin‐Stevens Point.

[eva70337-bib-0059] Krueger, C. C. , D. L. Perkins , E. L. Mills , and J. E. Marsden . 1995. “Predation by Alewives on Lake Trout Fry in Lake Ontario: Role of an Exotic Species in Preventing Restoration of a Native Species.” Journal of Great Lakes Research 21, no. 4: 458–469. 10.1016/S0380-1330(95)71117-0.

[eva70337-bib-0060] Kuparinen, A. , and S. Uusi‐Heikkilä . 2020. “Atlantic Cod Recovery From the Allee Effect Zone: Contrasting Ecological and Evolutionary Rescue.” Fish and Fisheries 21, no. 5: 916–926. 10.1111/FAF.12470.

[eva70337-bib-0061] Lachance, J. , and S. A. Tishkoff . 2013. “SNP Ascertainment Bias in Population Genetic Analyses: Why It Is Important, and How to Correct It.” BioEssays 35, no. 9: 780–786. 10.1002/bies.201300014. PMC3849385 23836388

[eva70337-bib-0062] Lachmuth, S. , W. Durka , and F. M. Schurr . 2011. “Differentiation of Reproductive and Competitive Ability in the Invaded Range of Senecio Inaequidens: The Role of Genetic Allee Effects, Adaptive and Nonadaptive Evolution.” New Phytologist 192, no. 2: 529–541. 10.1111/J.1469-8137.2011.03808.X. 21736567

[eva70337-bib-0063] Langlois, T. H. 1954. The Western End of Lake Erie and Its Ecology. J. W. Edwards.

[eva70337-bib-0064] Lawrence, M. , W. Huber , H. Pagès , et al. 2013. “Software for Computing and Annotating Genomic Ranges.” PLoS Computational Biology 9, no. 8: e1003118. 10.1371/journal.pcbi.1003118. PMC3738458 23950696

[eva70337-bib-0065] Li, H. 2013. “Aligning Sequence Reads, Clone Sequences and Assembly Contigs With BWA‐MEM.” arXiv Preprint. 10.48550/arXiv.1303.3997.

[eva70337-bib-0066] Lower, E. , R. Sturtevant , S. Iott , et al. 2024. “The Great Lakes' Most Unwanted: Characterizing the Impacts of the Top Ten Great Lakes Aquatic Invasive Species.” Journal of Great Lakes Research 50, no. 2: 102365. 10.1016/j.jglr.2024.102365.

[eva70337-bib-0067] MacDougall, T. , J. Buskiewicz , L. Sumner , and J. Schmitt . 2023. “2022 Report of the Lake Erie Coldwater Task Group.” Standing Technical Committee, Lake Erie Committee, Great Lakes Fishery Commission.

[eva70337-bib-0068] Malomane, D. K. , C. Reimer , S. Weigend , A. Weigend , A. R. Sharifi , and H. Simianer . 2018. “Efficiency of Different Strategies to Mitigate Ascertainment Bias When Using SNP Panels in Diversity Studies.” BMC Genomics 19: 22. 10.1186/s12864-017-4416-9. PMC5756397 29304727

[eva70337-bib-0069] Marie, A. D. , L. Bernatchez , and D. Garant . 2010. “Loss of Genetic Integrity Correlates With Stocking Intensity in Brook Charr ( *Salvelinus fontinalis* ).” Molecular Ecology 19, no. 10: 2025–2037. 10.1111/J.1365-294X.2010.04628.X. 20406382

[eva70337-bib-0070] Mason, N. , M. Ward , J. E. M. Watson , O. Venter , and R. K. Runting . 2020. “Global Opportunities and Challenges for Transboundary Conservation.” Nature Ecology & Evolution 4, no. 5: 694–701. 10.1038/s41559-020-1160-3. 32203481

[eva70337-bib-0071] Matthews, J. A. , and K. R. Briffa . 2005. “The ‘Little Ice Age’: Re‐Evaluation of an Evolving Concept.” Geografiska Annaler: Series A, Physical Geography 87, no. 1: 17–36. 10.1111/j.0435-3676.2005.00242.x.

[eva70337-bib-0072] McCairns, R. J. S. , A. Kuparinen , B. Panda , E. Jokikokko , and J. Merilä . 2012. “Effective Size and Genetic Composition of Two Exploited, Migratory Whitefish ( *Coregonus lavaretus lavaretus* ) Populations.” Conservation Genetics 13, no. 6: 1509–1520. 10.1007/S10592-012-0394-2.

[eva70337-bib-0073] McKenna, J. E. 2019. “The Laurentian Great Lakes: A Case Study in Ecological Disturbance and Climate Change.” Fisheries Management and Ecology 26, no. 5: 486–499. 10.1111/fme.12317.

[eva70337-bib-0074] McKinney, G. J. , C. E. Pascal , W. D. Templin , et al. 2020. “Dense SNP Panels Resolve Closely Related Chinook Salmon Populations.” Canadian Journal of Fisheries and Aquatic Sciences 77, no. 3: 451–461. 10.1139/cjfas-2019-0067.

[eva70337-bib-0075] Mérot, C. , K. S. R. Stenløkk , C. Venney , et al. 2023. “Genome Assembly, Structural Variants, and Genetic Differentiation Between Lake Whitefish Young Species Pairs (*Coregonus* sp.) With Long and Short Reads.” Molecular Ecology 32: 1458–1477. 10.1111/MEC.16468. 35416336

[eva70337-bib-0076] Miller, R. R. , J. D. Williams , and J. E. Williams . 1989. “Extinctions of North American Fishes During the Past Century.” Fisheries 14, no. 6: 22–38.

[eva70337-bib-0077] Nathan, L. R. , B. L. Sloss , J. A. VanDeHey , et al. 2016. “Temporal Stability of Lake Whitefish Genetic Stocks in Lake Michigan.” Journal of Great Lakes Research 42, no. 2: 433–439. 10.1016/j.jglr.2016.01.006.

[eva70337-bib-0078] Nei, M. , T. Maruyama , and R. Chakraborty . 1975. “The Bottleneck Effect and Genetic Variability in Populations.” Evolution 29, no. 1: 1–10. 10.2307/2407137. 28563291

[eva70337-bib-0079] Oldenburg, K. , M. A. Stapanian , P. A. Ryan , and E. Holm . 2007. “Potential Strategies for Recovery of Lake Whitefish and Lake Herring Stocks in Eastern Lake Erie.” Journal of Great Lakes Research 33, no. 1: 46–58. 10.3394/0380-1330(2007)33[46:psfrol]2.0.co;2.

[eva70337-bib-0080] Purcell, S. , B. Neale , K. Todd‐Brown , et al. 2007. “PLINK: A Tool Set for Whole‐Genome Association and Population‐Based Linkage Analyses.” American Journal of Human Genetics 81, no. 3: 559–575. 10.1086/519795. PMC1950838 17701901

[eva70337-bib-0081] R Core Team . 2025. R: A Language and Environment for Statistical Computing. R Foundation for Statistical Computing.

[eva70337-bib-0082] Rahman, A. , T. Q. Smith , and Z. A. Szpiech . 2025. “Fast and Memory‐Efficient Dynamic Programming Approach for Large‐Scale EHH‐Based Selection Scans.” Molecular Biology and Evolution 42: msaf275. 10.1093/MOLBEV/MSAF275. PMC12659807 41140120

[eva70337-bib-0083] Rahman, A. , T. Q. Smith , and Z. A. Szpiech . 2026. “Selection Scans and Downstream Analysis With Selscan.” Human Population Genetics and Genomics 6, no. 2: 0005. 10.47248/HPGG2606020005. PMC13411074 42524436

[eva70337-bib-0084] Regier, H. A. , V. C. Applegate , R. A. Ryder , et al. 1969. “The Ecology and Management of the Walleye in Western Lake Erie (Technical Report 15).” Great Lakes Fishery Commission.

[eva70337-bib-0085] Regier, H. A. , T. H. Whillans , W. J. Christie , and S. A. Bocking . 1999. “Over‐Fishing in the Great Lakes: The Context and History of the Controversy.” Aquatic Ecosystem Health & Management 2, no. 3: 239–248. 10.1080/14634989908656959.

[eva70337-bib-0086] Rogers, S. M. , N. Isabel , and L. Bernatchez . 2007. “Linkage Maps of the Dwarf and Normal Lake Whitefish ( *Coregonus clupeaformis* ) Species Complex and Their Hybrids Reveal the Genetic Architecture of Population Divergence.” Genetics 175: 375–398. 10.1534/GENETICS.106.061457. PMC1774998 17110497

[eva70337-bib-0087] Roseman, E. F. , G. W. Kennedy , J. Boase , B. A. Manny , T. N. Todd , and W. Stott . 2007. “Evidence of Lake Whitefish Spawning in the Detroit River: Implications for Habitat and Population Recovery.” Journal of Great Lakes Research 33, no. 2: 397–406. 10.1016/S0380-1330(07)70064-3.

[eva70337-bib-0088] Sadler, D. E. , P. C. Watts , and S. Uusi‐Heikkilä . 2023. “The Riddle of How Fisheries Influence Genetic Diversity.” Fishes 8, no. 10: 510. 10.3390/FISHES8100510.

[eva70337-bib-0122] Salzburger, W. , B. Van Bocxlaer , and A. S. Cohen . 2014. “Ecology and Evolution of the African Great Lakes and Their Faunas.” Annual Review of Ecology, Evolution, and Systematics 45, no. 1: 519–545. 10.1146/annurev-ecolsys-120213-091804.

[eva70337-bib-0089] Santiago, E. , C. Köpke , and A. Caballero . 2025. “Accounting for Population Structure and Data Quality in Demographic Inference With Linkage Disequilibrium Methods.” Nature Communications 16, no. 1: 1–10. 10.1038/s41467-025-61378-w. PMC12218012 40592851

[eva70337-bib-0090] Santiago, E. , I. Novo , A. F. Pardiñas , M. Saura , J. Wang , and A. Caballero . 2020. “Recent Demographic History Inferred by High‐Resolution Analysis of Linkage Disequilibrium.” Molecular Biology and Evolution 37, no. 12: 3642–3653. 10.1093/molbev/msaa169. 32642779

[eva70337-bib-0091] Schaefer, H. M. , A. E. Honsey , D. B. Bunnell , et al. 2022. “Predicting Physical and Geomorphic Habitat Associated With Historical Lake Whitefish and Cisco Spawning Locations in Lakes Erie and Ontario.” Journal of Great Lakes Research 48, no. 6: 1636–1646. 10.1016/j.jglr.2022.08.014.

[eva70337-bib-0092] Schaefer, H. M. , E. F. Roseman , R. L. DeBruyne , C. S. Vandergoot , and J. S. Diana . 2021. “Historic Coregonine Habitat Use and Assessment of Larval Nursery Locations in Lake Erie.” Advances in Limnology 66: 245–259. 10.1127/adv_limnol/2021/0072.

[eva70337-bib-0093] Schneider, J. C. , and J. H. Leach . 1977. “Walleye ( *Stizostedion vitreum vitreum* ) Fluctuations in the Great Lakes and Possible Causes, 1800–1975.” Journal of the Fisheries Research Board of Canada 34, no. 10: 1878–1889. 10.1139/f77-254.

[eva70337-bib-0094] Seaborn, T. , D. Griffith , A. Kliskey , and C. C. Caudill . 2021. “Building a Bridge Between Adaptive Capacity and Adaptive Potential to Understand Responses to Environmental Change.” Global Change Biology 27, no. 12: 2656–2668. 10.1111/gcb.15579. 33666302

[eva70337-bib-0095] Shi, Y. , J. J. Homola , P. T. Euclide , et al. 2022. “High‐Density Genomic Data Reveal Fine‐Scale Population Structure and Pronounced Islands of Adaptive Divergence in Lake Whitefish ( *Coregonus clupeaformis* ) From Lake Michigan.” Evolutionary Applications 15, no. 11: 1776–1791. 10.1111/eva.13475. PMC9679245 36426119

[eva70337-bib-0096] Stepien, C. A. , and J. E. Faber . 1998. “Population Genetic Structure, Phylogeography and Spawning Philopatry in Walleye ( *Stizostedion vitreum* ) From Mitochondrial DNA Control Region Sequences.” Molecular Ecology 7: 1757–1769. 10.1046/j.1365-294x.1998.00512.x. 9859203

[eva70337-bib-0097] Stott, W. , R. DeBruyne , and E. Roseman . 2024. “Genetic Origins of a Resurging Lake Whitefish, *Coregonus clupeaformis* , Population in the Detroit River, Laurentian Great Lakes.” International Journal of Limnology 60: 10. 10.1051/limn/2024010.

[eva70337-bib-0098] Stott, W. , M. P. Ebener , L. Mohr , T. Hartman , J. Johnson , and E. F. Roseman . 2013. “Spatial and Temporal Genetic Diversity of Lake Whitefish ( *Coregonus clupeaformis* (Mitchill)) From Lake Huron and Lake Erie.” Advances in Limnology 64: 205–222. 10.1127/1612-166X/2013/0064-0015.

[eva70337-bib-0099] Stott, W. , T. N. Todd , and L. Kallemeyn . 2004. “Genetic Variability Among Lake Whitefish From Isle Royale and the Upper Great Lakes.” Annales Zoologici Fennici 41, no. 1: 51–59.

[eva70337-bib-0100] Stott, W. , J. A. VanDeHey , and B. L. Sloss . 2010. “Genetic Diversity of Lake Whitefish in Lakes Michigan and Huron; Sampling, Standardization, and Research Priorities.” Journal of Great Lakes Research 36, no. S1: 59–65. 10.1016/j.jglr.2010.01.004.

[eva70337-bib-0101] Stow, C. A. , M. D. Rowe , C. M. Godwin , et al. 2023. “Lake Erie Hypoxia Spatial and Temporal Dynamics Present Challenges for Assessing Progress Toward Water Quality Goals.” Journal of Great Lakes Research 49: 981–992. 10.1016/J.JGLR.2023.02.008.

[eva70337-bib-0102] Szpiech, Z. A. 2024. “Selscan 2.0: Scanning for Sweeps in Unphased Data.” Bioinformatics 40, no. 1: btae006. 10.1093/bioinformatics/btae006. PMC10789311 38180866

[eva70337-bib-0103] Szpiech, Z. A. , T. E. Novak , N. P. Bailey , and L. S. Stevison . 2021. “Application of a Novel Haplotype‐Based Scan for Local Adaptation to Study High‐Altitude Adaptation in Rhesus Macaques.” Evolution Letters 5: 408–421. 10.1002/EVL3.232. PMC8327953 34367665

[eva70337-bib-0104] Tengstedt, A. N. B. , S. Liu , M. W. Jacobsen , et al. 2025. “Genomic Insights on Conservation Priorities for North Sea Houting and European Lake Whitefish (*Coregonus* spp.).” Molecular Ecology 34, no. 23: e17367. 10.1111/MEC.17367. PMC12684370 38686435

[eva70337-bib-0105] Tjeng, B. , M. Arimond , H. Bråten Grindeland , A. Dalla Libera , and A. Fulgione . 2025. “ParaMask: A New Method to Identify Multicopy Genomic Regions, Corrects Major Biases in Whole‐Genome Sequencing Data.” Genome Biology 26, no. 1: 20. 10.1186/s13059-025-03836-8. PMC12551310 41137035

[eva70337-bib-0106] Van der Auwera, G. , and B. O'Connor . 2020. Genomics in the Cloud: Using Docker, GATK, and WDL in Terra. 1st ed. O'Reilly Media.

[eva70337-bib-0107] Van der Auwera, G. A. , M. O. Carneiro , C. Hartl , et al. 2013. “From FastQ Data to High‐Confidence Variant Calls: The Genome Analysis Toolkit Best Practices Pipeline.” Current Protocols in Bioinformatics 43, no. 1: 11.10.1–11.10.33. 10.1002/0471250953.bi1110s43. PMC4243306 25431634

[eva70337-bib-0108] van Oosten, J. , and R. Hile . 1949. “Age and Growth of the Lake Whitefish, *Coregonus clupeaformis* (Mitchill), in Lake Erie.” Transactions of the American Fisheries Society 77: 178–249.

[eva70337-bib-0109] VanDeHey, J. A. , B. L. Sloss , P. J. Peeters , and T. M. Sutton . 2009. “Genetic Structure of Lake Whitefish ( *Coregonus clupeaformis* ) in Lake Michigan.” Canadian Journal of Fisheries and Aquatic Sciences 66, no. 3: 382–393. 10.1139/F08-213.

[eva70337-bib-0110] Vonlanthen, P. , D. Bittner , A. G. Hudson , et al. 2012. “Eutrophication Causes Speciation Reversal in Whitefish Adaptive Radiations.” Nature 482, no. 7385: 357–362. 10.1038/nature10824. 22337055

[eva70337-bib-0111] Vuorinen, J. A. , R. A. Bodaly , J. D. Reist , L. Bernatchez , and J. J. Dodson . 1993. “Genetic and Morphological Differentiation Between Dwarf and Normal Size Forms of Lake Whitefish ( *Coregonus clupeaformis* ) in Como Lake, Ontario.” Canadian Journal of Fisheries and Aquatic Sciences 50, no. 1: 210–216. 10.1139/f93-023.

[eva70337-bib-0112] Waples, R. S. 1998. “Separating the Wheat From the Chaff: Patterns of Genetic Differentiation in High Gene Flow Species.” Journal of Heredity 89, no. 5: 438–450. 10.1093/jhered/89.5.438.

[eva70337-bib-0113] Waples, R. S. , and O. Gaggiotti . 2006. “What Is a Population? An Empirical Evaluation of Some Genetic Methods for Identifying the Number of Gene Pools and Their Degree of Connectivity.” Molecular Ecology 15, no. 6: 1419–1439. 10.1111/j.1365-294X.2006.02890.x. 16629801

[eva70337-bib-0114] Waples, R. S. , A. E. Punt , and J. M. Cope . 2008. “Integrating Genetic Data Into Management of Marine Resources: How Can We Do It Better?” Fish and Fisheries 9, no. 4: 423–449. 10.1111/j.1467-2979.2008.00303.x.

[eva70337-bib-0115] Weir, B. S. , and C. C. Cockerham . 1984. “Estimating F‐Statistics for the Analysis of Population Structure.” Evolution 38, no. 6: 1358–1370. 10.2307/2408641. 28563791

[eva70337-bib-0116] Wickham, H. 2009. ggplot2: Elegant Graphics for Data Analysis. Springer‐Verlag New York. 10.1007/978-0-387-98141-3.

[eva70337-bib-0117] Wittmann, M. J. , W. Gabriel , and D. Metzler . 2014. “Population Genetic Consequences of the Allee Effect and the Role of Offspring‐Number Variation.” Genetics 198, no. 1: 311–320. 10.1534/genetics.114.167569. PMC4174943 25009148

[eva70337-bib-0118] Xiao, N. 2018. “ggsci: Scientific Journal and Sci‐Fi Themed Color Palettes for ‘ggplot2’ (R Package Version 2.9).” https://CRAN.R‐project.org/package=ggsci.

[eva70337-bib-0119] Ying, Y. , Y. Chen , L. Lin , and T. Gao . 2011. “Risks of Ignoring Fish Population Spatial Structure in Fisheries Management.” Canadian Journal of Fisheries and Aquatic Sciences 68, no. 12: 2101–2120. 10.1139/F2011-116.

[eva70337-bib-0120] Zeileis, A. , C. Kleiber , K. Walter , and K. Hornik . 2003. “Testing and Dating of Structural Changes in Practice.” Computational Statistics & Data Analysis 44: 109–123. 10.1016/S0167-9473(03)00030-6.

[eva70337-bib-0121] Zeileis, A. , F. Leisch , K. Hornik , and C. Kleiber . 2002. “strucchange: An R Package for Testing for Structural Change in Linear Regression Models.” Journal of Statistical Software 7: 1–38. 10.18637/JSS.V007.I02.

