# Supplementary material for: Genetic Bottleneck and Panmixia Following the Collapse of a Great Lakes Lake Whitefish Fishery
Source: Evol Appl. 2026 Oct 20;19(10):e70337. doi: 10.1111/eva.70337 (PMC13681039; doi:10.1111/eva.70337)
Supplement: Supplementary file 1 [file EVA-19-e70337-s003.docx]

Supplementary Materials

Figure S1

**Supplemental Figure S1**. Exact collection locations for N=371 Lake Whitefish collected for the present study. Genetic samples were collected from Lake Whitefish in the Detroit River and the west, central, and east basins of Lake Erie.

**Supplemental Figure S2**: Summary of whole genome sequencing dataset containing 127 lake whitefish and 5,444,182 retained SNP loci including (A) percent missing data by average depth of coverage (DOC) for each individual, (B) distribution of locus average DOC across all samples where the red line indicates the mean DOC, (C) distribution of Hardy-Weinberg equilibrium 1-tailed test for heterozygosity excess, and (C) final distribution of allele frequencies of the reference allele (black) and alternate allele (red).

**Supplemental Figure S3.** Commercial harvest data for Lake Whitefish in Lake Erie spanning 1867 – 2020. Commercial harvest of Lake Whitefish crashed in the late 1950s after reaching an annual high of just over 7 million lbs.

**Supplemental Figure S4**: Principal component analysis (PCA) biplots of the first to axes using only SNPs found within outlier windows detected among Lake Huron and Niagara and Toussaint Reefs (A, B), and among Lake Erie spawning site collections (C, D).
